# Scale effects on efficiency and profitability in the Swiss banking sector

**DOI:** 10.1186/s41937-022-00091-7

**Published:** 2022-05-09

**Authors:** Marc Blatter, Andreas Fuster

**Affiliations:** 1 Swiss National Bank, Börsenstrasse 15, 8022 Zurich, Switzerland; 2 EPFL, Swiss Finance Institute and CEPR, Lausanne, Switzerland

**Keywords:** Bank efficiency, Profitability, Economies of scale, Financial regulation, G21, G28

## Abstract

This paper analyzes efficiency and profitability in the Swiss banking sector over the period 1997–2019. We find strong evidence for scale economies: for most banks in the sample, efficiency and profitability increase with bank size. Using an instrumental variables strategy for a subset of geographically restrained banks, we find that the effect of size on efficiency and profitability is likely causal. Scale economies have been more pronounced since 2010 than in the years prior to the global financial crisis. There is little evidence for scale economies for the largest (systemically important) banks; their relatively lower efficiency and lower profitability appear driven by certain aspects of their business model. Our results further indicate that good capitalization and high efficiency and profitability are compatible.

**Supplementary Information:**

The online version contains supplementary material available at 10.1186/s41937-022-00091-7.

## Introduction

In recent years, the banking sector has faced important challenges. For instance, the globally low interest rates have gone hand in hand with reduced profitability, especially for banks that are more reliant on maturity transformation and net interest income (Claessens et al. 2018; Chaudron 2018; Molyneux et al. 2019). Furthermore, the digital transformation has already enhanced competition in the area of financial services, as FinTech and BigTech firms have entered the market, and this trend is expected to continue. The COVID-19 crisis comes on top of these pre-existing challenges to banks’ traditional business model, thereby putting additional pressure on the banking sector (Carletti et al. 2020). Aside from increasing the risk from nonperforming loans, the crisis has also accelerated digitalization tendencies, with increased popularity of contactless payments and FinTech apps used, for instance, for online trading.^1^

In such an environment, the efficiency of banking services is of particular relevance. Although an increase in competition due to digitalization and new entrants is likely beneficial from the perspective of consumers, the additional pressure on already historically low margins may have detrimental effects on the stability of the financial system (Swiss National Bank , 2019, p. 6). More efficient banks are more resilient against adverse market developments and in a better position to deal with increased competition. To anticipate the effects of the ongoing developments on the banking sector it is important to understand why some banks perform better than others. This naturally raises the question of whether bank size matters for efficiency.

Economies of scale in banking exist if the cost of producing an additional unit of a banking service (e.g., opening a bank account or providing a loan) decreases as the quantity of the service increases. The idea is that large banks can spread their overhead costs, such as information technology, accounting, advertising and personnel expenses, over a larger asset base. This would then increase profitability and make banks more economically viable and resilient against competition from new entrants, also providing them with the necessary room to make investments in new technologies. At the same time, an increase in bank size may also itself generate an increase in costs, for instance by enhancing organizational complexity.

Using data from 1997 to 2019, we analyze how efficiency and profitability metrics of Swiss banks depend on bank size. We report five main findings. First, looking simply at the time-series evolution of the efficiency and profitability measures, it is indeed the case that Swiss banks as a group have become less efficient and profitable over time, especially in the wake of the global financial crisis (GFC) a decade ago. Second, there is strong evidence for scale economies in a sample that excludes the largest, systemically important banks (SIBs): the cost-to-income ratio (CIR) decreases with bank size while the return on assets (ROA) increases with bank size. Over the sample period as a whole, the magnitude of the effect is economically modest: a one-standard-deviation increase in bank size is associated with a 2.1 percentage point lower CIR and a 0.06 percentage point higher ROA. However, our third finding is that scale efficiencies are more pronounced in the decade after the GFC than was the case over 1997–2006; for instance, a one-standard-deviation increase in bank size is associated with a 4 percentage point lower CIR in the more recent period. Adding various bank characteristics as control variables tends to further strengthen the estimated scale effects, at least for the CIR. Fourth, there is little evidence that the scale efficiencies extend to larger banks. The three domestic and two global Swiss SIBs in fact have significantly higher CIRs and lower ROAs than the largest non-SIBs. This may be due to the differences in the business model and balance sheet composition between the largest banks and their smaller counterparts—the globally active banks’ international scope and the relatively high share of trading assets seem to make it difficult for them to realize scale economies. Our fifth main finding is that good capitalization and high efficiency and profitability are compatible.

A thorny question for studies of scale economies is whether the relationship between size and efficiency is causal: are banks more efficient because they are larger, or are they larger because they are more efficient (and therefore able to grow)? We attempt to shed some light on this issue by relying on a subset of “cantonal banks” that historically operated almost exclusively within the boundaries of their home canton (and to this day do most of their business in that canton).^2^ As a consequence, the size of these banks is strongly correlated with the population of their home canton. We therefore use the population of cantons in 1995 as an instrumental variable (IV) for bank size. OLS and IV estimates turn out to be similar within this sample, and confirm the presence of scale economies, especially in the post-GFC period. This exercise thus suggests that there is likely a causal effect of bank size on efficiency/profitability.

Our evidence for economies of scale in the banking sector suggests that small- and medium-sized banks will face particular challenges in an environment characterized by reduced profitability and increased competition. Consolidation via mergers could be a natural way to increase efficiency. A merger may lead to cost savings, for instance, by reducing overlaps in the branch network and by consolidating back-office work. A recent example for a big domestic merger is that between the Spanish banks CaixaBank and state-owned Bankia announced in September 2020. It is to be expected that similar deals will follow elsewhere in the banking union. In fact, the European regulator is encouraging new alliances. The European Central Bank (ECB) recently published a guideline outlining its supervisory approach to consolidation in the banking sector and announced that it will make use of its supervisory tools to facilitate sustainable consolidation projects.^3^

Our paper relates to an extensive empirical literature on efficiency and scale economies in banking. Overall, the evidence on scale economies is not fully conclusive. Early studies using data on US banks found economies of scale limited to relatively small banks with deposits from $10–25 million (Benston et al. 1982) or assets less than $10 billion (Berger and Mester 1997).^4^ More recent studies instead find evidence for economies of scale for larger banks (Wheelock and Wilson 2012; Kovner et al. 2014; Hughes et al. 2019). A possible explanation for why optimal bank size may have increased is the dissemination of information technologies and the proliferation of scalable market-based operations (Laeven et al. 2014). Wheelock and Wilson (2018) analyze the evolution of scale economies in the US banking sector and find that most of the largest US banks had increasing returns to scale both before and after the financial crisis. For European banks, the evidence is mixed. Beccalli et al. (2015) find evidence for economies of scale across different size classes of banks, including the biggest banks. In contrast, for banks in postwar Germany, Huber (2020) finds that banks did not become more efficient or more profitable after increasing in size.

Like most studies on economies of scale, we focus on cost economies, i.e., the ability of banks to efficiently use overhead in administrative and back-office operations. To estimate scale economies, we follow the methodology used by Bertay et al. (2013) and Kovner et al. (2014). Basically, this consists of regressing common measures for efficiency and profitability on bank size, controlling for various bank characteristics. Like Hughes et al. (2019), we also form different bank size categories and examine cost and revenue differences across those categories. The advantage of a methodology relying on common efficiency measures is that it is transparent and easy to replicate, therefore allowing for a comparison of results from different countries. Many other studies, e.g., Berger and Mester (1997) and Wheelock and Wilson (2018), use alternative (and more complicated) methodologies based on the parametric or nonparametric estimation of bank cost (and also revenue and profit) functions. From those, they derive estimates of returns to scale.

There are a few previous studies on efficiency in the Swiss banking sector. Using estimated cost and profit functions, Rime and Stiroh (2003) examined the performance of Swiss banks in the 1996–1999 period. They found evidence of economies of scale for small and mid-size banks, but not for the largest banks. Our sample includes fewer banks (since we restrict the sample to domestically focused banks), but for a much longer period, 1997–2019. In the middle of our sample period, the Swiss banking sector was hit by the 2007–2009 financial crisis, making necessary a public intervention to stabilize the largest Swiss bank. After a period of sustained growth since the late 1990s, the large, universal banks have substantially reduced their total assets in recent years. Overall, our results confirm the evidence for scale economies in Rime and Stiroh (2003), but only for the later years. In the early part of our sample period, we do not find much evidence that our efficiency and profitability metrics improve with bank size, even for small to mid-size banks.

Dietrich and Wanzenried (2011) use a dynamic panel approach to analyze the determinants of bank profitability for Swiss banks in the 1999–2009 period. Using the Fitch-IBCA Bankscope database, they find that bank profitability is mainly explained by operational efficiency, the growth of total loans, funding costs and the business model. They use the CIR as a measure of operational efficiency and the ROA as a measure of profitability, but do not focus on bank size as a determinant of these outcomes. They find that more efficient banks are more profitable than less efficient banks.^5^

We contribute to the literature on efficiency in banking by using a sample with a long time-series dimension, covering more than 20 years and allowing us to differentiate between the period before and after the GFC. The sample includes banks which differ substantially with regard to their size and business model (in particular, the non-SIBs versus the two globally active banks, UBS and Credit Suisse). While it is difficult to fully disentangle the importance of size versus other bank characteristics in explaining efficiency and profitability differences across these bank types, we propose an intuitive methodology for doing so. Furthermore, to our knowledge, we are the first to instrument for bank size based on the (historical) population of the local area where banks conduct most of their business.

From a social welfare perspective, besides scale economies, the efficient scale of banks also depends on other factors that we do not consider in this paper. In particular, we do not directly consider economies of diversification or scope, i.e., the returns when banks can use information from one activity to offer other activities at lower costs (e.g., Drucker and Puri 2005). Neither do we consider potential costs of banks being too-big-to-fail (TBTF). Hughes and Mester (2013) note that a larger bank size may generate scale economies due to diversification, but also due to incentives to take more risk. In a model accounting for risk-taking, they find large-scale economies for US banks, which are not driven by TBTF subsidies. In contrast, Davies and Tracey (2014) no longer find evidence of scale economies for a sample of large banks after controlling for TBTF factors.

To determine policy implications, it is necessary to balance the efficiency gains from larger banks against the potential reduction in bank competition and a potential increase in financial stability risks. Boyd and Heitz (2016) try to balance the social cost and benefits of TBTF banks. They conclude that the potential benefits due to economies of scale are unlikely to exceed the potential costs due to increased systemic risk.^6^ Assessing the benefits of scale efficiencies arising from larger banks against reduced bank competition is particularly challenging. The social welfare effects of bank competition itself are ambiguous. Competition is a driver of efficiency, but it may also be detrimental for financial stability due to excessive risk-taking or credit expansion (see Vives , 2019). Due to the complicated relationship between competition and financial stability in banking, we do not attempt to draw policy recommendations concerning the socially optimal bank size.

The rest of this paper is organized as follows. Section 2 provides an overview of the Swiss banking sector. Section 3 describes the dataset, defines the efficiency measures used to test for economies of scale and provides descriptive statistics. Sections 4 and 5 present and discuss the results. Section 6 concludes.

## The Swiss banking sector

The banking sector plays an important role in Switzerland’s economy.^7^ Total banking sector assets amount to around 500% of Swiss GDP, which is high by international standards. Banks are the main providers of essential financial services such as domestic deposit taking and lending; there is relatively little nonbank credit intermediation.^8^ The banking sector accounts for about 5% of value added in Switzerland.

Banks in Switzerland differ significantly with regard to their size, business model, geographical scope of activities and legal form. For the purpose of our analysis, the Swiss banking sector can be broken down into three main categories: (i) the two globally active banks, Credit Suisse and UBS, (ii) the domestically focused banks, primarily comprising regional, cantonal and Raiffeisen^9^ banks and (iii) other banks, including domestic banks as well as branches and subsidiaries of foreign banks.

The business models of the three bank categories are very different. The two globally active banks, Credit Suisse and UBS, are universal banks with a large proportion of foreign business. They put a special focus on international wealth management, but they also have substantial activities in domestic deposit and lending business as well as investment banking. The domestically focused banks concentrate on deposit and lending business, with a special focus on mortgage lending. Most banks in the “other banks” category focus on wealth management.

Of the 237 banks in Switzerland, the SNB has designated five banks as SIBs. These include the two globally active banks, Credit Suisse and UBS (G-SIBs), and three domestically focused banks, Zürcher Kantonalbank (ZKB), Raiffeisen Group and PostFinance (D-SIBs). The Swiss banking sector is characterized by the dominance of a small number of banks. Together, the five systemically important banks account for more than half of the domestic deposit and lending business. The other domestically focused banks account for roughly one-third. The market share of the “other banks” category is less than one-tenth.

## Data and descriptive statistics

We rely on data from statistical surveys of banks carried out by the Swiss National Bank. In order to ensure the confidentiality of information provided by individual institutions, the Swiss National Bank only publishes aggregated data. For our analysis, we rely on individual bank data over the period 1997–2019. We use consolidated data, with a few exceptions.^10^ As the main sample for our analysis, we take banks that are classified as “domestically focused,” meaning that the banks have a share of domestic loans to total assets exceeding 50% or play a prominent role in the domestic deposit market. In addition, we include the largest banks (Credit Suisse and UBS, and their predecessors). Even though these two globally active universal banks have a large proportion of foreign business (roughly 70% of their respective balance sheets), they also have substantial activities in domestic deposit and lending markets.

Our sample restriction means that we do not include wealth management banks or private banks in our main analysis. The reason for this is that these types of banks have a different business model, with little focus on domestic deposit taking and lending. Hence, these banks’ revenue structure is also different: instead of interest income, fee and commission income is the dominant component of their total income. Nevertheless, given that wealth management is a core business for both universal banks, we include wealth management banks as an additional comparison group when assessing scale effects for G-SIBs in Sect. 4.3. Our sample restriction further excludes foreign-controlled banks and branches of foreign banks operating in Switzerland. Those banks, which also primarily focus on wealth management, are often not legal entities in their own right but part of their foreign parent company. Furthermore, their reported income is likely to heavily depend on transactions with the parent company.

**Fig. 1:**
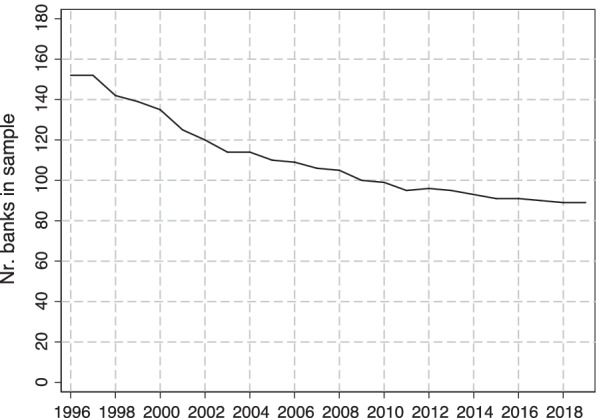
Number of banks in main analysis sample, by year

The number of banks in the sample over time is shown in Fig. 1. We see that there is a decrease over time, which is particularly rapid in the late 1990s/early 2000s. Exits from the sample can occur for different reasons: banks can be acquired (or merge with other banks); they can be reclassified; or they may no longer be required to report because their size falls below the reporting threshold. In Additional file 1: Appendix B, we explicitly study exits from the sample that occur through acquisition.

Our main outcome variables of interest are the following:

1 $$\begin{aligned} \mathrm{Cost}\ \mathrm{income}\ \mathrm{ratio}\ (\mathrm{CIR})&= \frac{\mathrm{operating}\ \mathrm{expense}}{\mathrm{operating}\ \mathrm{income}} \end{aligned}$$

2 $$\begin{aligned} \mathrm{Return} \ \mathrm{on} \ \mathrm{assets}\ (\mathrm{ROA})&= \frac{\mathrm{net}\ \mathrm{income}}{\mathrm{total}\ \mathrm{assets}}. \end{aligned}$$

The CIR is a measure of banks’ efficiency—the lower this ratio, the more efficient the bank. Operating expense in the numerator is the sum of personnel expense and material expense. Operating income in the denominator is the sum of net interest income, net commission and services income, net trading income, investment income and net other ordinary income. The ROA is a measure of bank profitability—a higher ratio indicates better profitability. Net income in the numerator captures the bank’s profits (or losses).

Both CIR and ROA are widely used in the literature and by financial analysts. Xu et al. (2019) provide stylized facts on CIRs and ROAs for several groups of banks in the period 2004–2017. ROAs declined sharply during the GFC and US banks recovered faster than European banks. In 2017, ROA was higher for US banks (0.8%) than for European banks (0.4%).^11^ The value for the group of G-SIBs was in between (0.6%). In general, the picture is symmetric for the CIR. CIRs recovered after the peak of the GFC. Between 2014 and 2017, cost efficiency improved for US banks and G-SIBs and stayed more or less constant for European banks. In 2017, European banks had a higher CIR (67%) than US banks (61%) and G-SIBs (56%). As a rule of thumb, banks with a CIR below 50% are considered efficient.^12^

When considering ROA, it is important to note that some banks manage securities holdings for their customers in bank custody accounts.^13^ There are important differences in such off-balance sheet activities across the banks in the sample. The globally active banks have a much higher share of securities holdings in bank custody accounts than non-systemically important banks. The largest non-SIBs have some off-balance sheet business, whereas the smallest non-SIBs do not (see Additional file 1: Appendix Figure A.1). The off-balance sheet business generates commission income but does not increase a bank’s assets. In that sense, one could view the ROA of banks with more off-balance sheet business as overstating profitability relative to total exposure, compared to banks with no or limited off-balance sheet business. We will take this into account by using a bank’s fraction of commission income over operating income as a control variable in our empirical analysis.

As additional efficiency measures, we consider:

3 $$\begin{aligned} \mathrm{Material}\ \mathrm{expense}\ \mathrm{income}\ \mathrm{ratio}\ (\mathrm{CIR}_{\mathrm{mat}})&= \frac{\mathrm{material}\ \mathrm{expense}}{\mathrm{operating}\ \mathrm{income}} \end{aligned}$$

4 $$\begin{aligned} \mathrm{Personnel}\ \mathrm{expense}\ \mathrm{income}\ \mathrm{ratio}\ (\mathrm{CIR}_{\mathrm{pers}})&= \frac{\mathrm{personnel}\ \mathrm{expense}}{\mathrm{operating}\ \mathrm{income}} \end{aligned}$$

5 $$\begin{aligned} \mathrm{Expense} \ \mathrm{assets}\ \mathrm{ratio}\ (\mathrm{EAR})&= \frac{\mathrm{noninterest}\ \mathrm{expense} }{\mathrm{total}\ \mathrm{assets}} \end{aligned}$$

The $\mathrm{CIR}_{\mathrm{mat}}$ and the $\mathrm{CIR}_{\mathrm{pers}}$ serve to decompose the cost income ratio into material costs (such as IT expenditures and rents) and personnel costs (wages). The EAR is an alternative efficiency measure, which is also used by Kovner et al. (2014). Noninterest expense is an area in which banks can potentially realize cost advantages from size. The division by total assets serves as an alternative normalization; total assets may be less volatile than operating income (the denominator in the CIR).

As additional profitability measures, we consider:

6 $$\begin{aligned} \mathrm{Net}\ \mathrm{operating} \ \mathrm{income} \ \mathrm{assets}\ \mathrm{ratio}\ (\mathrm{NOI/TA})&= \frac{\mathrm{operating}\ \mathrm{income} - \mathrm{operating}\ \mathrm{expense} }{\mathrm{total}\ \mathrm{assets}} \end{aligned}$$

7 $$\begin{aligned} \mathrm{Return} \ \mathrm{on} \ \mathrm{risk}\ \mathrm{weighted}\ \mathrm{assets}\ (\mathrm{RORWA})&= \frac{\mathrm{net}\ \mathrm{income}}{\mathrm{risk}\ \mathrm{weighted}\ \mathrm{assets}} \end{aligned}$$

8 $$\begin{aligned} \mathrm{Return} \ \mathrm{on} \ \mathrm{equity}\ (\mathrm{ROE})&= \frac{\mathrm{net}\ \mathrm{income}}{\mathrm{total}\ \mathrm{equity}} \end{aligned}$$

The difference between the ROA and the NOI/TA is the measure of income in the numerator. ROA uses net income, i.e., profits or losses, whereas NOI/TA only considers net operating income, which does not include taxes and provisions for loan losses that might distort net income. The two measures, RORWA and ROE, differ from ROA with respect to the denominator. RORWA uses risk-weighted assets instead of total assets, which should therefore account for the possibility that some banks are more profitable due to higher risk-taking (which ROA would not account for). ROE is a measure of profitability from the equity holder perspective, which is commonly used as a complement to ROA.^14^

Our main explanatory variable is bank size, which we measure by (the logarithm of) total assets. Other variables such as total deposits, the number of clients or the number of employees could also serve as a proxy for bank size. The total assets measure has the advantage that it depends less on a particular bank’s business and funding model than these alternatives. It is also by far the most common measure used in the related literature.

In our regressions, we will control for various bank characteristics. First, we capture a bank’s business model by the share of deposits, mortgages and trading assets over total assets. Second, we consider the bank’s revenue structure by the share of net interest income, commission income and trading income over operating income. Third, we consider the bank’s risk profile with the capital over assets ratio and the RWA density. Fourth, we include measures for concentration of the bank’s business, namely the share of domestic assets over total assets and a Herfindahl–Hirschman Index (HHI) capturing the regional diversification of the bank’s mortgages across Swiss cantons. Finally, we include another HHI capturing the average canton-level mortgage market concentration across the cantons a bank is active in (weighted by the outstanding mortgage amounts the bank has in each canton) as a proxy for its market power. Additional file 1: Appendix Figure A.2 shows the time-series evolution of these variables.

Importantly, we observe balance sheet variables at year-end only, while income and expense variables are totals over the course of a year. When we use bank assets either as our explanatory variable of interest or to normalize other variables, we first take the average of years $t-1$ and *t*. Furthermore, any control variables other than size are taken as of year-end $t-1$. Thus, even though our data go back to 1996, the first year of the sample is 1997. We winsorize all variables at the 1st and 99th percentile (over the entire sample), except log(Total Assets). Summary statistics for the different outcome and control variables are provided in Table 1.

**Table 1:** Descriptive statistics

	Mean	SD	Min	Median	Max	N
Cost/income ratio (%)	54.26	10.28	32.37	53.55	87.25	2497
Return on assets (%)	0.37	0.19	− 0.06	0.34	1.33	2497
Personnel cost/income (%)	30.69	7.10	17.18	29.94	56.92	2497
Material cost/income (%)	23.57	6.56	11.51	22.89	44.24	2497
Expense/asset ratio (%)	1.10	0.37	0.59	1.04	2.94	2497
Net operating income/assets (%)	0.58	0.31	− 0.62	0.56	1.75	2497
Return on risk-wtd. assets (%)	0.69	0.38	− 0.09	0.61	2.63	2345
Return on equity (%)	4.81	2.61	− 1.73	4.43	18.07	2497
Log(Total Assets)	20.89	2.08	15.40	20.37	28.48	2497
Deposits/assets (%)	63.90	9.34	39.34	63.96	90.22	2497
Mortgages/assets (%)	73.60	13.49	10.08	77.38	88.03	2497
Trading/assets (%)	0.74	2.82	0.00	0.03	20.85	2497
Net Int. Inc./Op. Inc. (%)	78.06	15.29	21.86	80.73	101.53	2497
Commission Inc./Op. Inc. (%)	15.90	12.24	0.00	13.09	63.21	2497
Trading Inc./Op. Inc. (%)	4.12	4.18	− 5.62	3.27	22.52	2497
Capital/assets (%)	7.95	2.06	3.59	7.67	14.84	2497
RWA/Assets (%)	54.50	8.44	25.31	54.63	81.99	2497
Domestic/total assets (%)	96.34	11.33	19.31	99.47	100.00	2497
HHI of Mtg Holdings (/1000)	7.50	2.52	0.82	8.37	10.00	1801
Avg. local HHI (/1000)	1.96	0.54	1.12	2.00	3.64	1801

Variable definitions are discussed in Sect. 3. “HHI of Mtg Holdings” is the Herfindahl–Hirschman Index of a bank’s mortgage holdings across the 26 Swiss cantons in a given year; it measures how locally concentrated a bank’s mortgage business is. “Avg. Local HHI” is based on each canton’s annual HHI (using the market shares of all banks active in a canton), and we then take a weighted average for each bank, using its share of mortgages across cantons as weights. It thus measures how concentrated the mortgage markets are where a bank operates (a proxy for market power). Both HHI measures are only available since 2002

**Fig. 2:**
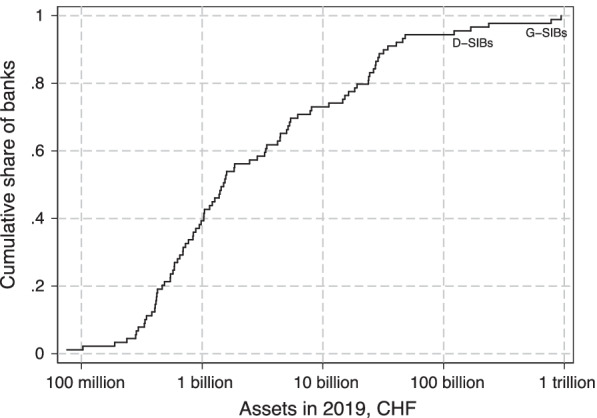
Cumulative distribution of bank size in 2019 among banks included in analysis sample. Notes: Figure shows the cumulative distribution of total assets in 2019 for banks included in our analysis sample, on a log scale. Total assets in 2019 are measured as the average of year-end assets in 2018 and 2019. The two largest banks are the G-SIBs Credit Suisse and UBS; the following three banks are the D-SIBs Zürcher Kantonalbank (ZKB), Raiffeisen Group and PostFinance. Data source: SNB

In our analysis, we will separately consider those banks classified as systemically important (SIBs). These are the two global SIBs (G-SIBs), UBS and Credit Suisse (and their respective predecessors), as well as the domestic SIBs (D-SIBs) Raiffeisen, PostFinance and ZKB.^15^ The main reason for separating them from the other banks in the sample is that they are substantially larger, as illustrated in Fig. 2. Furthermore, due to their systemic importance, they are subject to special regulatory requirements.^16^ In Additional file 1: Appendix Table A.1, we show descriptive statistics separately for the SIB and non-SIB samples.

**Fig. 3:**
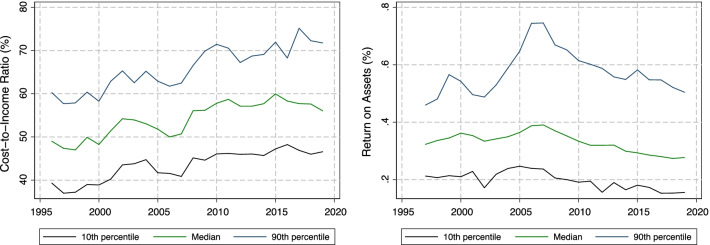
Evolution of CIR and ROA in sample over time. Notes: Figure shows the evolution of median, 10th percentile and 90th percentile across banks in our sample of cost income ratio (CIR) in the left panel and return on assets (ROA) in the right panel. Data source: SNB.

The time series of the median CIR and ROA, as well as their 10th and 90th percentiles, are shown in Fig. 3. We note that CIRs trended upward over the 1995–2010 period, while being approximately flat since then. ROAs reached their highest levels in 2006–2007, but have since been steadily declining. Compared to the benchmarks given above, efficiency appears relatively low (the median CIR has exceeded 50% in all years since 2001), and the same is true for profitability, especially when ROAs are compared to US banks.^17^

**Fig. 4:**
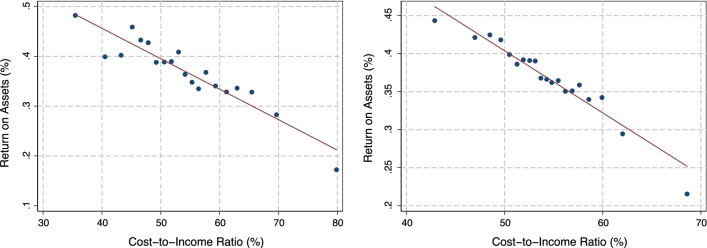
Relationship between ROA and CIR. Note: Binned scatter plots. Left panel: no controls (pooled sample); right panel: controlling for bank and year fixed effects

Figure 4 shows the relationship between CIR and ROA, in order to illustrate that the efficiency measure (CIR) does strongly correlate with the profitability measure (ROA). The figure consists of binned scatter plots, where the variable on the horizontal axis is grouped into 20 bins, and the corresponding average values for the variable on the vertical axis are plotted. In the left panel, this is done for the pooled sample across all years (without control variables). The right panel repeats this but controls for year fixed effects (so that annual averages are subtracted from both CIRs and ROAs) and bank fixed effects (so that deviations of bank values from their respective averages are used). The relationship remains essentially equally strong in the right panel, meaning that within a bank, decreases in the CIR and increases in ROA occur simultaneously, suggesting that indeed, an improvement in measured efficiency improves bank profitability.

## Results

### Scale effects for non-SIBs

Table 2 presents results of simple linear regressions of CIR or ROA on Log(Total Assets) for the non-SIB sample. In all regressions, we control for year fixed effects, meaning that we exploit only cross-sectional variation. In columns (1) and (5), we use the full sample, covering 1997–2019 and a total of 148 banks. We find significant evidence for scale efficiencies: the CIR decreases with bank size, while ROA increases in bank size.^18^ The magnitude of the effects appears modest: for instance, the coefficients imply that a 10% increase in bank size is associated with a 0.12 percentage point lower CIR and a 0.004 percentage point higher ROA. However, it is worth bearing in mind that the bank sizes, even in our non-SIB sample, are very heterogeneous: the within-year standard deviation of log(Total Assets) is about 1.7, so that a one-standard-deviation increase in size lowers the CIR by 2.1 percentage points, corresponding to about 0.2 standard deviations of the CIR. Similarly, a one-standard-deviation increase in size increases ROA by 0.06 percentage points, corresponding to about 0.4 standard deviations of ROA.

**Table 2:** Regressions of CIR (%) and ROA (%) on log(Total Assets) for non-SIBs, without additional controls

	(1)	(2)	(3)	(4)	(5)	(6)	(7)	(8)
	CIR	CIR	CIR	CIR	ROA	ROA	ROA	ROA
Log(Total Assets)	$-$1.243***	$-$0.332	$-$2.337***	$-$15.097**	0.036***	0.018**	0.054***	0.038
	(0.391)	(0.403)	(0.469)	(5.787)	(0.007)	(0.007)	(0.009)	(0.069)
Year FE?	Yes	Yes	Yes	Yes	Yes	Yes	Yes	Yes
Bank FE?	No	No	No	Yes	No	No	No	Yes
Years	All years	1997–2006	2010–2019	2010–2019	All years	1997–2006	2010–2019	2010–2019
Nr. banks	148	146	96	92	148	146	96	92
N	2390	1211	881	877	2390	1211	881	877
Mean(dep. var.)	53.71	50.47	57.86	57.83	0.37	0.37	0.34	0.34
SD(dep. var.)	9.95	9.07	9.47	9.46	0.19	0.19	0.17	0.17
Adj. R2	0.20	0.07	0.17	0.80	0.13	0.04	0.28	0.83
Adj. R2 (within)	0.05	0.00	0.17	0.04	0.11	0.03	0.27	$-$0.00

Robust standard errors (clustered by bank) in parentheses.

Significance: *$$<0.1$$, *$$<0.05$$, ***$$<0.01$$

Columns (2) and (3) for CIR and (6) and (7) for ROA show how scale efficiencies differ across pre- vs. post-GFC samples. The results indicate much stronger scale efficiencies since 2010 than in the decade prior to the crisis, when there was no significant relationship between bank size and the CIR, and only a small positive relationship of bank size and ROA. In the decade after the GFC, a 10% increase in bank size is associated with a 0.23 percentage point lower CIR and a 0.005 percentage point higher ROA. We also note that in the post-GFC period, size differences across banks explain a non-trivial share of the variation in CIR and ROA within-year: the adjusted $R^2$ is 0.17 in column (3) and 0.27 in column (7).

**Fig. 5:**
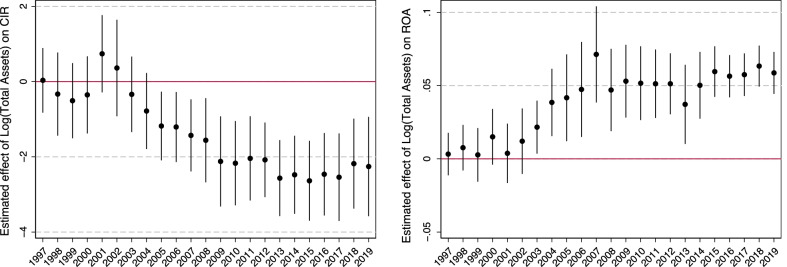
Evolution of estimated relationship between log(Total Assets) and efficiency/profitability. Note: Plots show estimated coefficients $\beta _t$ from regressions $y_{it}=\alpha _t + \beta _t log(Total Assets)_{it} + \varepsilon _{it}$, with associated 95% confidence intervals (standard errors clustered by bank). In the left panel, *y* is the cost-to-income ratio (CIR); in the right panel, *y* is return on assets (ROA)

The time-variation in estimated scale effects is further illustrated in Fig. 5, which shows the evolution of annual cross-sectional coefficients on log(Total Assets), along with 95% confidence intervals. The left panel shows that there was no significant relationship between size and the CIR prior to 2005, and the estimated coefficient then becomes more negative again after 2008. For ROA, the relationship with size emerged a bit sooner, as shown in the right panel; the estimated coefficient has been significantly positive and relatively stable since 2004, with one outlier to the upside (2007) and one to the downside (2013).

Finally, columns (4) and (8) of Table 2 add bank fixed effects, focusing only on the post-crisis period. That is, the regression coefficients in these columns capture the relationship between CIR (or ROA) and log(Total Assets) *within bank*. In column (4), this increases the coefficient more than sixfold—a 10% increase in a bank’s size is now associated with a 1.5 percentage point decrease in the CIR.^19^ In contrast, the coefficient for ROA in column (8) does not change much, but is very imprecisely estimated.

In Tables 3 and 4, we add various bank characteristics to the regressions, to see how they correlate with CIR and ROA (conditional on bank size) and also to test whether they affect the estimated relationship between the efficiency and profitability measures and size itself. We proceed by adding different “blocks” of variables one-by-one on the right-hand-side. These variables often may capture the same underlying characteristic, e.g., differences in bank business models. Thus, we generally do not want to put too much weight on the significance of individual coefficients, although to the extent that they are stable across specifications and subsamples (pre- vs. post-crisis), it makes it more likely that there may be a “structural” relationship.

**Table 3:** Regressions of CIR on log(Total Assets) and controls, for non-SIBs

	(1)	(2)	(3)	(4)	(5)	(6)	(7)
Log(Total Assets)	$-$1.513***	$-$2.100***	$-$2.018***	$-$2.715***	$-$3.017***	$-$2.165***	$-$4.179***
	(0.373)	(0.404)	(0.387)	(0.565)	(0.378)	(0.367)	(0.608)
Deposits/Assets (%)	$-$0.041				0.037	0.066	0.005
	(0.098)				(0.086)	(0.087)	(0.101)
Mortgages/Assets (%)	$-$0.102				$-$0.091	$-$0.160*	0.004
	(0.083)				(0.092)	(0.088)	(0.132)
Trading/Assets (%)	0.268				0.018	$-$0.277	0.124
	(0.492)				(0.264)	(0.340)	(0.644)
Net Int. Inc./Op. Inc. (%)		$-$0.082			$-$0.107	$-$0.044	$-$0.151
		(0.097)			(0.091)	(0.128)	(0.118)
Commission Inc./Op. Inc. (%)		0.189*			0.193*	0.218	0.130
		(0.105)			(0.099)	(0.139)	(0.147)
Trading Inc./Op. Inc. (%)		$-$0.218			$-$0.266*	$-$0.173	$-$0.296
		(0.161)			(0.140)	(0.157)	(0.271)
Capital/Assets (%)			$-$1.375***		$-$1.780***	$-$1.633***	$-$1.670***
			(0.320)		(0.309)	(0.302)	(0.365)
RWA/Assets (%)			0.367***		0.225***	0.276***	0.125
			(0.084)		(0.084)	(0.095)	(0.108)
Domestic/Total Assets (%)				$-$0.301			$-$0.198
				(0.251)			(0.311)
HHI of Mtg Holdings (/1000)				$-$1.043**			$-$0.758*
				(0.425)			(0.443)
Avg. Local HHI (/1000)				$-$0.235			$-$0.066
				(1.100)			(1.256)
Year FE?	Yes	Yes	Yes	Yes	Yes	Yes	Yes
Bank FE?	No	No	No	No	No	No	No
Years	All	All	All	All	All	1997–2006	2010–2019
Nr. banks	148	148	139	111	139	137	96
N	2386	2386	2242	1607	2242	1064	880
Mean(dep. var.)	53.72	53.72	54.14	55.78	54.14	50.90	57.89
SD(dep. var.)	9.93	9.93	9.88	9.56	9.88	9.06	9.44
Adj. R2	0.21	0.24	0.27	0.24	0.33	0.21	0.37
Adj. R2 (within)	0.07	0.10	0.16	0.17	0.23	0.17	0.37

Robust standard errors (clustered by bank) in parentheses.

Significance: *$$<0.1$$, *$$<0.05$$, ***$$<0.01$$

**Table 4:** Regressions of ROA on log(Total Assets) and controls, for non-SIBs

	(1)	(2)	(3)	(4)	(5)	(6)	(7)
Log(Total Assets)	0.026***	0.006	0.048***	0.036***	0.026***	0.007	0.042***
	(0.007)	(0.009)	(0.007)	(0.011)	(0.007)	(0.008)	(0.008)
Deposits/Assets (%)	0.000				0.001	0.001	0.000
	(0.002)				(0.001)	(0.001)	(0.001)
Mortgages/Assets (%)	$-$0.006**				$-$0.001	0.001	$-$0.003*
	(0.002)				(0.002)	(0.002)	(0.002)
Trading/Assets (%)	$-$0.007				$-$0.021***	$-$0.016	$-$0.034***
	(0.008)				(0.007)	(0.010)	(0.008)
Net Int. Inc./Op. Inc. (%)		$-$0.005***			$-$0.004***	$-$0.005***	0.001
		(0.002)			(0.001)	(0.002)	(0.001)
Commission Inc./Op. Inc. (%)		0.003*			0.003**	0.003*	0.006***
		(0.002)			(0.001)	(0.002)	(0.002)
Trading Inc./Op. Inc. (%)		$-$0.003			$-$0.001	$-$0.003	0.005
		(0.004)			(0.003)	(0.003)	(0.004)
Capital/Assets (%)			0.045***		0.038***	0.038***	0.033***
			(0.008)		(0.005)	(0.006)	(0.005)
RWA/Assets (%)			$-$0.001		$-$0.004**	$-$0.003	$-$0.003**
			(0.002)		(0.002)	(0.002)	(0.001)
Domestic/Total Assets (%)				$-$0.017***			0.001
				(0.006)			(0.004)
HHI of Mtg Holdings (/1000)				0.015**			0.007
				(0.006)			(0.006)
Avg. Local HHI (/1000)				0.057***			0.041**
				(0.016)			(0.016)
Year FE?	Yes	Yes	Yes	Yes	Yes	Yes	Yes
Bank FE?	No	No	No	No	No	No	No
Years	All	All	All	All	All	1997–2006	2010–2019
Nr. banks	148	148	139	111	139	137	96
N	2386	2386	2242	1607	2242	1064	880
Mean(dep. var.)	0.37	0.37	0.37	0.37	0.37	0.38	0.34
SD(dep. var.)	0.18	0.18	0.19	0.19	0.19	0.18	0.17
Adj. R2	0.21	0.28	0.36	0.34	0.44	0.36	0.61
Adj. R2 (within)	0.18	0.26	0.33	0.31	0.42	0.34	0.61

Robust standard errors (clustered by bank) in parentheses.

Significance: *$$<0.1$$, *$$<0.05$$, ***$$<0.01$$

In the first five columns of the tables, we use data from the full 1997–2019 period. However, as the data underlying the two HHI measures are only available since 2002, the regressions in column (4) only start in 2003 (given that independent variables are lagged by one year). For this reason, when we combine all independent variables in columns (5) and (6), we exclude the last block of independent variables.

Table 3 indicates a strong relationship between banks’ capital ratios and their CIR: banks with more capital are relatively more efficient (they have a lower CIR). Aside from this strongly significant relationship, there are a few slightly less robust correlations: higher RWA density tends to be associated with a higher CIR (which could be due to increased screening and monitoring needs for riskier assets), while more locally concentrated mortgage holdings are associated with a lower CIR, which is intuitive. Across the full sample, there are also marginally significant effects of the shares of income coming from commissions and trading.

Importantly, comparing columns (5) through (7) to their counterparts in Table 2 shows that when controls are added, the estimated relationship between the CIR and bank size, and thus the evidence for scale efficiencies, becomes stronger. This implies that larger banks tend to have characteristics that are associated with a higher CIR; therefore, not controlling for these characteristics attenuates the estimated relationship between size and the CIR.

In Table  4, we turn to ROA as the dependent variable. We again find a strong association with capital ratios: controlling for size, banks with higher capital ratios have higher ROA, meaning they are more profitable. Of course, we cannot attach a causal interpretation to this relationship; it may simply reflect that profitable banks “naturally” build up capital by not paying out all their profits. Nevertheless, the strength of the relationship at least casts doubt on the possibility of a causal relationship going in the opposite direction (i.e., that holding more capital would reduce ROA).^20^

Aside from the capital ratio, other significant variables (at least in some columns) include the share of assets in mortgages or trading assets, the share of income coming from net interest income or commission income, and the RWA density. Note that a high share of commission income increases ROA. This is in line with our hypothesis that ROA of banks with more off-balance sheet business is likely to be biased upward compared to banks with no or only a small amount of off-balance sheet business (see discussion in Sect. 3). The last row of the table indicates that banks that are mostly active in more concentrated local markets (cantons) tend to have higher ROA, suggesting they may have more market power.

Unlike for the CIR, adding the additional controls does not strengthen the relationship between ROA and size; if anything, the coefficient on log(Total Assets) is smaller than in Table 2. Nevertheless, at least for the full sample and the post-GFC sample, the relationship between size and ROA remains positive and strongly significant.

A potential factor that could affect estimated scale effects in the Swiss context is that many non-SIBs collaborate in networks in some way, primarily with the goal of sharing some of the operational costs.^21^ The networks assist member banks in back-office operations (such as IT management, legal and compliance). In particular, networks provide member banks with a common IT solution for banking services and offer centralized support. Moreover, networks facilitate member banks’ access to money and capital markets. Furthermore, the cantonal banks may benefit not only from some pooled activities, but also from the state (cantonal) guarantees that apply for almost all of them. This may increase ROA in particular, by reducing funding costs relative to other non-SIBs.

In Additional file 1: Appendix Table A.2, we control directly for network membership and cantonal bank dummies to see to what extent this affects estimated scale efficiencies. Without other control variables, adding the cantonal bank and network member dummies reduces the estimated coefficients on log(Total Assets) and in case of the CIR over the full sample period renders it statistically insignificant. This may partly reflect the strong collinearity between size and cantonal bank status in this sample. However, once other control variables are added, the estimated size effect is only slightly smaller than in the corresponding regressions in Tables 3 and 4. Thus, it does not appear that size effects are primarily picking up cantonal bank status or network membership.^22^ Looking at the coefficients on these dummy variables directly, it does appear that both types of banks benefit in terms of a lower CIR. However, there is little benefit in terms of ROA; in fact, banks that are network members have significantly lower ROA. We further study scale effects within cantonal banks directly in the next subsection.

### Is the effect of bank size causal?

In the analysis above, we found a strong relationship between bank size, CIR and ROA, especially in the post-GFC period. An obvious question is to what extent the observed relationship reflects a *causal* link from bank size to efficiency or profitability. We already discussed that adding various control variables tends to strengthen the estimated effect of size, which suggests that size is not just a proxy for other aspects of a bank’s business model that might lead to higher efficiency or profitability (if anything, the opposite seems to be true). However, there could still be factors that are unobservable to us that might drive both size and efficiency. One example could be management quality: good management might achieve better efficiency and be able to attract additional funding, thereby growing the bank. This is a general problem for the literature on bank scale economies: it is difficult to find plausibly exogenous variation in bank size that would allow one to estimate a causal effect on efficiency and profitability.^23^

In the Swiss market, we study, there exist some banks whose size is arguably to a large extent exogenously determined: the so-called cantonal banks, of which there are 24. Historically, these banks did almost all of their business in their home canton; even now, most of their lending is still in the home canton.^24^ As a consequence, the size of cantonal banks is highly positively correlated with the canton’s population, for instance, in 2019, the simple correlation between log(Total Assets) of the cantonal bank and log(canton population) was +0.88. This suggests that we can compare the efficiency and profitability of cantonal banks across cantons of different sizes—or econometrically, use cantonal population as an instrument for bank size. Furthermore, rather than using contemporaneous population, we can use the population from before the start of our sample period (1995), so it is not affected by economic developments during the sample period.^25^

Population is a valid instrument for bank size if it fulfills the exclusion restriction: it should only affect efficiency or profitability of the local cantonal bank through its effect on the bank’s size. The exclusion restriction would be invalid if population size also proxies for other local characteristics that could simultaneously affect these outcomes—for instance, the productivity of the local labor force. While it is difficult to fully rule out such a channel, in the regressions below we control for the share of the local population with completed tertiary education (which indeed tends to be higher in larger cantons than in the smallest, rural cantons). However, there is at most limited evidence that this productivity proxy affects the CIR and ROA of the local cantonal bank, and adding this control variable leaves the coefficients on instrumented bank size almost unchanged.

Results comparing OLS (as above) and 2SLS (with log cantonal population as of 1995 as excluded instrument for log(Total Assets)) are shown in Table 5, in panel A for the pre-crisis period 1997–2006 and in panel B for the post-crisis period 2010–2019. As in the earlier results, scale economies for cantonal banks are weaker in the pre-crisis period and, at most, marginally significant. In panel B, however, we see significant evidence of scale economies, especially when control variables are added (in columns 3–4 for CIR and 7–8 for ROA). The OLS coefficients can be compared to those in columns (7) of Tables 3 and 4 (which feature the same bank characteristics as control variables); scale effects on the CIR seem slightly weaker for cantonal banks than for all non-SIBs, while scale effects on ROA are stronger.

**Table 5:** Cantonal banks: instrumenting size by local population

	(1)	(2)	(3)	(4)	(5)	(6)	(7)	(8)
	CIR	CIR	CIR	CIR	ROA	ROA	ROA	ROA
*A. Pre-crisis period (1997–2006)*								
Log(Total Assets)	0.953	1.972	$-$1.438	$-$1.036	0.042	0.027	0.060*	0.064*
	(1.299)	(1.253)	(1.271)	(1.370)	(0.025)	(0.024)	(0.034)	(0.033)
Pop. share w/tert. educ.	$-$13.934	$-$23.466	$-$23.182	$-$24.962	$-$0.297	$-$0.160	$-$0.510	$-$0.530
	(35.802)	(33.769)	(20.006)	(20.575)	(0.542)	(0.526)	(0.323)	(0.337)
Method	OLS	IV	OLS	IV	OLS	IV	OLS	IV
Controls?	No	No	Yes	Yes	No	No	Yes	Yes
Year FE?	Yes	Yes	Yes	Yes	Yes	Yes	Yes	Yes
Nr. banks	24	24	24	24	24	24	24	24
N	240	240	240	240	240	240	240	240
Adj. R2	0.04		0.48		0.29		0.49	
First-stage F-stat		127.6		74.6		127.6		74.6

	(1)	(2)	(3)	(4)	(5)	(6)	(7)	(8)
	CIR	CIR	CIR	CIR	ROA	ROA	ROA	ROA
*B. Post-crisis period (2010–2019)*								
Log(Total Assets)	$-$0.390	1.224	$-$3.745***	$-$3.286**	0.078***	0.062**	0.111***	0.091***
	(1.690)	(1.566)	(1.291)	(1.503)	(0.027)	(0.023)	(0.021)	(0.023)
Pop. share w/tert. educ.	9.370	$-$5.145	$-$5.958	$-$7.833	$-$0.595	$-$0.445	$-$0.394*	$-$0.312
	(23.904)	(22.727)	(11.422)	(11.840)	(0.358)	(0.340)	(0.208)	(0.199)
Method	OLS	IV	OLS	IV	OLS	IV	OLS	IV
Controls?	No	No	Yes	Yes	No	No	Yes	Yes
Year FE?	Yes	Yes	Yes	Yes	Yes	Yes	Yes	Yes
Nr. banks	24	24	24	24	24	24	24	24
N	240	240	240	240	240	240	240	240
Adj. R2	$-$0.04		0.55		0.22		0.59	
First-stage F-stat		110.6		342.2		110.6		342.2

	(1)	(2)	(3)	(4)				
	1997–2006		2010–2019					
*C. First-stage regressions for IV specifications (dep. var.: Log(Total Assets))*								
Log(Canton Population in 1995)	0.699***	0.668***	0.689***	1.046***				
	(0.062)	(0.077)	(0.066)	(0.057)				
Pop. share w/tert. educ.	3.872***	3.258**	3.599**	2.153***				
	(1.031)	(1.181)	(1.379)	(0.673)				
Controls?	No	Yes	No	Yes				
Year FE?	Yes	Yes	Yes	Yes				
Nr. banks	24	24	24	24				
N	240	240	240	240				
Adj. R2	0.87	0.92	0.86	0.96				

Robust standard errors (clustered by bank) in parentheses.

Significance: *$$<0.1$$, *$$<0.05$$, ***$$<0.01$$

More importantly, the comparison of the matching OLS and IV columns in Table 5 indicates that the IV coefficients are only slightly smaller than the OLS coefficients, suggesting at most a limited bias from potential omitted variables in the OLS estimation. The IV estimates in panel B indicate significant causal effects of bank size on the CIR (when other bank controls are added) and ROA (with and without other controls).^26^

We also note that the share of the population with tertiary education in a canton at most seems to have a small effect on cantonal banks’ efficiency and profitability; in fact, for ROA, the point estimate is negative.^27^ Finally, panel C of Table 5 shows the results from the first-stage regressions of the IV analysis, where we find that the estimated elasticity of log(Total Assets) on log(Cantonal Population) is roughly between 0.7 and 1.0 (depending on the period and on whether other bank characteristics are controlled for), meaning that across cantons, a 10% difference in population in 1995 is associated with a 7–10% difference in cantonal bank assets. The strong relationship between the variables is also reflected in the high first-stage F-statistics displayed in panels A and B.

In sum, if one accepts that the (past) size of a canton’s population is a valid instrumental variable for the size of its cantonal bank, this analysis implies a significant causal effect of bank size on the CIR and ROA in the post-crisis period: at least in the period since 2010, larger cantonal banks have tended to exhibit lower CIRs and higher ROAs *because* they are larger.

### Do SIBs also exhibit scale efficiencies?

So far, we have found evidence for scale efficiencies in a sample of domestically focused Swiss banks that are not deemed systemically important. In this section, we attempt to shed light on the question of whether these scale efficiencies continue to manifest for larger banks. This issue is challenging to study, since the largest Swiss banks, UBS and Credit Suisse, in particular have rather different characteristics from the banks in the non-SIB sample that we have focused on so far. Thus, if we observe differential efficiency or profitability, it is difficult to know to what extent this can be explained by size itself, versus differences in other characteristics. This concern is especially pertinent for characteristics where there is little overlap between the non-SIB and SIB samples; then, adding this characteristic as a control is essentially collinear with a G-SIB dummy. We present an attempt to deal with this, although we caution against putting too much weight on the results in this section.

Rather than postulating that CIR and ROA should move linearly with log(Total Assets), in this section we instead divide non-SIBs into five equal-sized groups (quintiles, formed by size within each year), and then add separate indicator variables for D-SIBs and G-SIBs. Thus, we study a total of seven groups of banks.

**Fig. 6:**
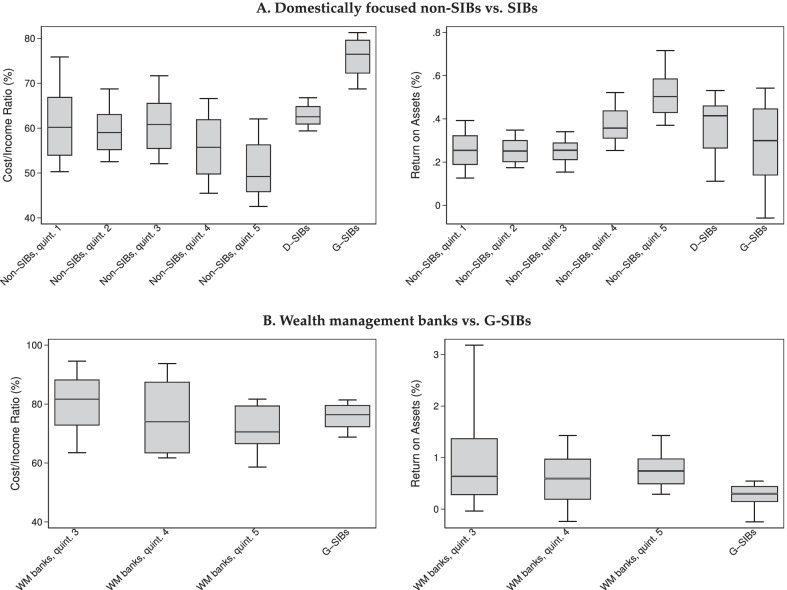
Unconditional variation of CIR and ROA across bank size groups. Note: box plots for pooled sample period 2010–2019. Boxes show 25th, 50th and 75th percentile; whiskers show 10th and 90th percentile. Size quintiles for non-SIBs and wealth management (WM) banks are defined by year. In panel B, only quintiles 3–5 are shown because smaller wealth management banks exhibit even higher variation in CIR and ROA

First, we provide graphical evidence on how efficiency and profitability vary across these bank groups, focusing on the period since 2010. Panel A of Fig. 6 shows for both CIR and ROA that, in line with what we saw above, efficiency and profitability tend to increase in size for domestically focused non-SIBs. However, we also note that D-SIBs tend to have higher CIRs and lower ROAs than the smaller banks, and the difference is even starker for G-SIBs.

Since the two G-SIBs have substantial activities in wealth management, we additionally compare their efficiency and profitability with those of Swiss wealth management banks. We only include the three largest quintiles of the wealth management banks, because CIRs and ROAs are very volatile in the two smallest quintiles (the corresponding banks are also very small measured by total assets). Panel B of Fig. 6 shows that for quintiles 3–5 of wealth management banks CIR tends to decrease in size, whereas ROA tends to increase in size. This evidence for positive scale effects is in line with our previous observation for other non-SIBs. As before, there is no evidence that these scale effects extend to G-SIBs; these banks tend to have higher CIRs and lower ROAs than the wealth management banks in size quintiles 4 and 5. However, at least for the CIR, the difference is less stark than in panel A.

Second, we turn to regressions, using indicators for the same groups of domestically focused banks as shown in panel A of Fig. 6 (five non-SIB groups, with the smallest one as omitted category, plus dummies for the three D-SIBs and the two G-SIBs) as main independent variables of interest. We estimate three different specifications (for both CIR and ROA), over the whole sample period and separately for the post-crisis years 2010-2019. First, we only add year fixed effects. Second, we add the full set of control variables as in Tables 3 and 4.^28^ Third, we include the control variables, but estimate their coefficients only on the non-SIB sample, and then constrain the coefficients to those values when estimating the group coefficients on the full sample (including the SIB observations). The idea behind this approach is that, as described above, separately estimating SIB effects while including all other controls is difficult because the G-SIBs in particular strongly differ in some dimensions from the non-SIBs. This third specification, then, essentially amounts to assuming that the relationship between controls and outcomes that is estimated for non-SIBs can be extrapolated to SIBs as well. In turn, this allows for a more precise estimate of the size effect itself for the SIBs.

Results are presented in Tables 6 and 7. Columns (1) and (4) confirm the graphical evidence from the box plots above: D-SIBs and especially G-SIBs have significantly higher CIRs and lower ROAs than the largest non-SIBs, especially in the post-crisis period. However, the other columns suggest that at least to some extent, these differences can be “explained away” by adding the other bank characteristics as controls. For instance, column (5) of Table 6 shows that when controls are added, the point estimate of the G-SIB dummy is $-12$, meaning that after accounting for bank characteristics, the G-SIB banks have lower CIRs than the omitted category (the smallest non-SIBs) and only slightly higher CIRs than the largest non-SIBs (with an estimated coefficient of $-17.4$). However, as expected, based on the discussion above, the G-SIB dummy is very imprecisely estimated, with a standard error of 14.5. Thus, the specification shown in this column does not allow one to draw any precise conclusions about the relative efficiency of G-SIBs. Once we constrain the coefficients on the control variables to be equal to those estimated in the non-SIB sample, however, precision improves drastically. As shown in column (6), the standard error on the G-SIB dummy is below 2, so that the coefficient of $-15.5$ now suggests that indeed, G-SIBs’ cost efficiency is close to that of the largest non-SIBs, even though the residual CIR remains slightly higher. Also of note, according to both columns (4) and (5), is that the D-SIBs have higher CIRs than the largest two quintiles of non-SIBs even after bank characteristics are controlled for.^29^

**Table 6:** Regressions of CIR on size group, D-SIB and G-SIB dummies and controls

	(1)	(2)	(3)	(4)	(5)	(6)
Non-SIB, size quintile 2	$-$4.115**	$-$5.314***	$-$5.339***	$-$2.055	$-$4.742**	$-$4.743**
	(1.663)	(1.346)	(1.542)	(2.333)	(1.835)	(1.983)
Non-SIB, size quintile 3	$-$2.327	$-$5.610***	$-$5.810***	$-$0.741	$-$4.899**	$-$4.835**
	(1.956)	(1.778)	(1.814)	(2.645)	(2.371)	(2.307)
Non-SIB, size quintile 4	$-$4.733**	$-$9.402***	$-$9.619***	$-$5.722**	$-$11.484***	$-$11.439***
	(2.052)	(1.812)	(1.714)	(2.747)	(2.563)	(2.151)
Non-SIB, size quintile 5	$-$6.775***	$-$13.489***	$-$13.634***	$-$10.676***	$-$17.375***	$-$17.203***
	(2.149)	(1.976)	(1.799)	(2.763)	(2.813)	(2.217)
D-SIB	1.739	$-$6.949**	$-$5.884***	1.668	$-$8.985**	$-$8.873***
	(1.788)	(2.750)	(1.623)	(2.413)	(3.836)	(1.798)
G-SIB	15.473***	$-$5.457	$-$0.658	14.368***	$-$12.049	$-$15.479***
	(2.527)	(5.706)	(2.916)	(2.060)	(14.525)	(1.939)
Controls?	No	Yes, unconstr.	Yes, constr.	No	Yes, unconstr.	Yes, constr.
Year FE?	Yes	Yes	Yes	Yes	Yes	Yes
Years	All	All	All	2010–2019	2010–2019	2010–2019
Banks	All	All	All	All	All	All
Nr. banks	155	145	145	101	101	101
N	2504	2343	2343	928	926	926
Mean(dep. var.)	54.27	54.68	54.68	58.41	58.43	58.43
SD(dep. var.)	10.27	10.20	10.20	9.69	9.66	9.66
Adj. R2	0.27	0.37	.36	0.23	0.36	.36

Robust standard errors (clustered by bank) in parentheses.

Significance: *$$<0.1$$, *$$<0.05$$, ***$$<0.01$$

**Table 7:** Regressions of ROA on size group, D-SIB and G-SIB dummies and controls

	(1)	(2)	(3)	(4)	(5)	(6)
Non-SIB, size quintile 2	$-$0.005	$-$0.015	$-$0.011	0.004	$-$0.006	0.001
	(0.024)	(0.020)	(0.020)	(0.032)	(0.019)	(0.018)
Non-SIB, size quintile 3	0.016	$-$0.000	0.004	0.011	$-$0.011	$-$0.009
	(0.036)	(0.023)	(0.022)	(0.035)	(0.022)	(0.019)
Non-SIB, size quintile 4	0.090***	0.038	0.040*	0.113***	0.055*	0.053**
	(0.031)	(0.026)	(0.023)	(0.034)	(0.031)	(0.022)
Non-SIB, size quintile 5	0.170***	0.126***	0.121***	0.253***	0.202***	0.184***
	(0.031)	(0.031)	(0.027)	(0.038)	(0.036)	(0.027)
D-SIB	0.129**	0.194***	0.170**	0.105	0.145*	0.106
	(0.058)	(0.071)	(0.085)	(0.076)	(0.076)	(0.142)
G-SIB	0.072	0.232	0.147**	0.007	0.438	0.482***
	(0.091)	(0.176)	(0.061)	(0.064)	(0.282)	(0.045)
Controls?	No	Yes, unconstr.	Yes, constr.	No	Yes, unconstr.	Yes, constr.
Year FE?	Yes	Yes	Yes	Yes	Yes	Yes
Years	All	All	All	2010–2019	2010–2019	2010–2019
Banks	All	All	All	All	All	All
Nr. banks	155	145	145	101	101	101
N	2497	2343	2343	927	926	926
Mean(dep. var.)	0.37	0.37	0.37	0.34	0.34	0.34
SD(dep. var.)	0.19	0.19	0.19	0.18	0.17	0.17
Adj. R2	0.15	0.43	.42	0.31	0.58	.54

Robust standard errors (clustered by bank) in parentheses.

Significance: *$$<0.1$$, *$$<0.05$$, ***$$<0.01$$

In Table 7, with ROA as the outcome, the effects of adding the controls are similarly large. In this case, in the post-crisis period, the G-SIB effect is strongly positive and exceeds the estimated effect for the largest non-SIBs by 0.23 and 0.30 percentage points in columns (5) and (6), respectively, compared to $-0.25$ percentage points in column (4). Thus, even though G-SIBs are unconditionally less profitable than non-SIBs in the top quintiles of the size distribution, conditional on their characteristics, they appear to be *more* profitable.

**Table 8:** Decomposing the effect of control variables on the estimated difference in CIR and ROA between largest non-SIB and G-SIBs

	(1)	(2)	(3)	(4)	(5)	(6)
	Mean Q5	Mean G-SIB	CIR, 2010–2019		ROA, 2010–2019	
			*Coefficient*	*Implied effect on G-SIB vs. Q5*	*Coefficient*	*Implied effect on G-SIB vs. Q5*
Deposits/Assets (%)	66.44	44.82	0.054	1.160	0.000	0.002
Mortgages/Assets (%)	69.01	14.45	0.012	0.654	− 0.002	− 0.118
Trading/Assets (%)	0.64	15.57	0.273	− 4.074	− 0.032	0.482
Net Int. Inc./Op. Inc. (%)	68.83	27.36	− 0.114	− 4.722	0.000	0.003
Commission Inc./Op. Inc. (%)	23.33	58.91	0.119	− 4.230	0.007	− 0.244
Trading Inc./Op. Inc. (%)	6.99	11.88	− 0.324	1.587	0.005	− 0.025
Capital/Assets (%)	8.17	4.85	− 1.379	− 4.581	0.027	0.090
RWA/Assets (%)	50.89	28.02	0.115	2.629	− 0.002	− 0.046
Domestic/Total Assets (%)	95.19	23.64	− 0.121	− 8.682	0.005	0.358
HHI of Mtg Holdings (/1000)	5.97	1.05	− 0.637	− 3.138	0.008	0.038
Avg. Local HHI (/1000)	1.99	1.90	0.726	0.060	0.035	0.003
Total				− 23.34		0.544

The first two columns show the average characteristics over 2010–2019 of non-SIBs in the largest quintile (Q5) vs. the G-SIBs. The third column shows the estimated coefficient on a given variable in the regression shown in column (6) of Table 6 (i.e., estimated on the non-G-SIB sample only). The fourth column then multiplies the difference between columns (1) and (2) with this coefficient from column (3). This yields the implied effect on non-SIB CIRs relative to G-SIBs’. The sum of these values adds up to the change in the gap between the Q5 and G-SIB coefficients between column (4) and column (6) of Table 6. The final two columns undertake a similar decomposition for the ROA regressions

Given the importance of the control variables for the conclusions that one draws from this analysis, it is worth decomposing their effects. We do so in Table 8, focusing on the 2010–2019 period. The first two columns show the average characteristics of non-SIBs in the largest quintile (Q5) vs. the G-SIBs. We note large differences in many dimensions; for instance, the G-SIBs hold a much lower share of their assets in mortgages (but more in trading assets), make more of their income through commissions and less through net interest income, hold less capital, have a lower risk density (RWA/Assets), and hold less than a quarter of their assets domestically. The third column then shows the estimated coefficient on a given variable in the regression shown in column (6) of Table 6 (i.e., estimated on the non-G-SIB sample only). For instance, a one percentage point higher Capital/Asset ratio is associated with a 1.379 percentage points lower CIR. The fourth column then multiplies the difference between columns (1) and (2) with this coefficient from column (3). This yields the implied effect on non-SIBs’ CIR relative to G-SIBs’. For the example of Capital/Assets, the fact that non-SIBs have an average ratio that is 3.32 ($=8.17-4.85$) percentage points higher “explains” a reduction in their CIR of 4.851 percentage points. When this is done for all characteristics, the total adds up to $-23.3$ percentage points, which is by how much the gap between the Q5 and G-SIB coefficients changes from column (4) to column (6) of Table 6.^30^ The final two columns of Table 8 undertake a similar decomposition for the ROA regressions.

There are several takeaways from this decomposition. For the CIR, the main differences in characteristics that “justify” why G-SIBs have a higher CIR are the following: first, their domestic asset share is much lower, and since higher domestic shares are associated with lower CIRs, that accounts for over one-third of their higher CIR relative to (domestically focused) large non-SIBs. After that, there are four other characteristics that account for the bulk of the remaining difference: the asset and revenue composition, and the difference in Capital/Assets. Similarly, for ROA (last column), by far the two most important “explanations” for why the unconditional ROA of G-SIBs is lower while after adding controls their dummy coefficient is positive and quite large is that they hold many more trading assets (which are associated with a lower ROA) and that their domestic asset share is low. A countervailing effect comes from the commission share of income, which is generally associated with a higher ROA. Controlling for the commission income over operating income share, we take into account that G-SIBs have more off-balance sheet business than non-SIBs, which, as discussed earlier, increases measured ROA for G-SIBs.

We emphasize that the decomposition we described is a purely mechanical exercise, and one cannot derive prescriptions for how any given bank could improve its CIR or ROA from our results. Nevertheless, they suggest that certain aspects of G-SIBs’ business models, such as their international diversification or the relatively high trading asset share, may make it difficult for them to realize scale efficiencies.

**Table 9:** Regressions of CIR and ROA on log(Assets) for SIBs, without additional controls

	(1)	(2)	(3)	(4)	(5)	(6)	(7)	(8)
	CIR	CIR	CIR	CIR	ROA	ROA	ROA	ROA
Log(Total Assets)	6.252***	6.313***	$-$3.155	1.718	$-$0.238***	$-$0.254***	0.242*	$-$0.125
	(1.326)	(1.441)	(3.463)	(2.985)	(0.074)	(0.080)	(0.138)	(0.169)
G-SIB	1.854	1.611	18.667***		0.402**	0.543***	$-$0.545**	
	(2.553)	(2.822)	(6.275)		(0.159)	(0.186)	(0.250)	
Year FE?	Yes	Yes	Yes	Yes	Yes	Yes	Yes	Yes
Bank FE?	No	No	No	Yes	No	No	No	Yes
Years	All years	1997–2006	2010–2019	2010–2019	All years	1997–2006	2010–2019	2010–2019
Nr. banks	7	6	5	5	7	6	5	5
N	107	49	46	46	107	49	46	46
Mean(dep. var.)	66.53	63.69	68.83	68.83	0.41	0.49	0.32	0.32
SD(dep. var.)	9.99	10.28	7.81	7.81	0.30	0.35	0.19	0.19
Adj. R2	0.63	0.57	0.68	0.71	0.32	0.38	0.10	0.46
Adj. R2 (within)	0.66	0.61	0.73	$-$0.03	0.18	0.23	0.11	$-$0.01

Robust standard errors in parentheses.

Significance: *$$<0.1$$, *$$<0.05$$, ***$$<0.01$$

Finally, Table 9 provides another test of scale effects, within the SIB-sample only. It shows the equivalent regressions to those in Table 2 but for SIBs only.^31^ In these regressions, we do not control for bank characteristics, although we do add a G-SIB dummy, to account for the fact that there are substantial differences in the business models of the mostly domestically focused D-SIBs and the globally active G-SIBs. Columns (1) and (5) show that within this group of banks, larger firms have higher CIR and lower ROA in the cross section when considering the full sample period. Interestingly, however, there are directional differences when looking separately before vs. after the financial crisis period; columns (3) and (7) show that since 2010, there is weak evidence for scale economies within D-SIB and G-SIB groups, although G-SIBs have substantially higher CIRs and lower ROAs. Columns (4) and (8) add bank fixed effects over this post-crisis period and find no evidence for scale economies; in fact, directionally, the CIR increases and ROA falls as a bank gets larger, even though statistical power is very limited.

### Alternative outcome measures

In the previous analysis, we used CIR and ROA as measures of efficiency and profitability. In this section, we consider additional measures of efficiency and profitability, which are defined in Sect. 3. To do so, we repeat the regressions from above that use simple indicators for different size groups (five non-SIB groups plus separate indicators for D-SIBs and G-SIBs). For simplicity, we focus on the specifications without control variables, except for year fixed effects.^32^ We conduct the analysis both over the whole sample and over the post-crisis period 2010–2019.

Results are shown in Table 10. Panel A focuses on alternative efficiency metrics, while panel B considers alternative profitability metrics.^33^ Columns (1) and (5) correspond to the specifications in columns (1) and (4) of Tables 6 and 7 and are shown again to provide a benchmark for the remaining columns.

**Table 10:** Alternative measures for efficiency and profitability

	(1)	(2)	(3)	(4)	(5)	(6)	(7)	(8)
	CIR	CIR$_{mat}$	CIR$_{pers}$	EAR	CIR	CIR$_{mat}$	CIR$_{pers}$	EAR
*Panel A*								
Non-SIB, size quintile 2	$-$4.115**	$-$3.227***	$-$0.841	$-$0.075	$-$2.055	$-$2.048	$-$0.051	0.053
	(1.663)	(1.008)	(1.319)	(0.058)	(2.333)	(1.465)	(1.984)	(0.081)
Non-SIB, size quintile 3	$-$2.327	$-$4.058***	1.756	0.019	$-$0.741	$-$3.654**	2.872	0.033
	(1.956)	(1.213)	(1.403)	(0.077)	(2.645)	(1.765)	(1.991)	(0.059)
Non-SIB, size quintile 4	$-$4.733**	$-$6.790***	2.109	0.013	$-$5.722**	$-$9.261***	3.529*	$-$0.061
	(2.052)	(1.248)	(1.449)	(0.072)	(2.747)	(1.714)	(2.014)	(0.056)
Non-SIB, size quintile 5	$-$6.775***	$-$8.594***	1.833	0.024	$-$10.676***	$-$10.918***	0.186	$-$0.061
	(2.149)	(1.297)	(1.505)	(0.067)	(2.763)	(1.898)	(1.900)	(0.065)
D-SIB	1.739	$-$6.550***	8.289***	0.072	1.668	$-$7.398*	9.018***	0.107
	(1.788)	(2.232)	(1.811)	(0.065)	(2.413)	(4.243)	(3.303)	(0.078)
G-SIB	15.473***	$-$6.291***	21.341***	1.129***	14.368***	$-$8.381***	22.381***	1.141***
	(2.527)	(1.290)	(2.270)	(0.089)	(2.060)	(1.372)	(1.836)	(0.091)
Controls?	No	No	No	No	No	No	No	No
Year FE?	Yes	Yes	Yes	Yes	Yes	Yes	Yes	Yes
Years	All	All	All	All	2010–2019	2010–2019	2010–2019	2010–2019
Banks	All	All	All	All	All	All	All	All
Nr. banks	155	155	155	155	101	101	101	101
N	2504	2504	2504	2497	928	928	928	927
Mean(dep. var.)	54.27	23.56	30.71	1.10	58.41	24.96	33.46	1.00
SD(dep. var.)	10.27	6.56	7.10	0.37	9.69	7.14	6.80	0.31
Adj. R2	0.27	0.25	0.34	0.28	0.23	0.33	0.28	0.37

	(1)	(2)	(3)	(4)	(5)	(6)	(7)	(8)
	ROA	NOI/TA	RORWA	ROE	ROA	NOI/TA	RORWA	ROE
**Panel B**								
Non-SIB, size quintile 2	$-$0.005	0.001	$-$0.039	0.143	0.004	$-$0.005	$-$0.010	0.427
	(0.024)	(0.035)	(0.051)	(0.269)	(0.032)	(0.044)	(0.059)	(0.319)
Non-SIB, size quintile 3	0.016	$-$0.003	$-$0.028	0.477	0.011	$-$0.004	$-$0.004	0.633
	(0.036)	(0.047)	(0.059)	(0.314)	(0.035)	(0.050)	(0.071)	(0.383)
Non-SIB, size quintile 4	0.090***	0.109**	0.117*	1.388***	0.113***	0.106**	0.188***	1.751***
	(0.031)	(0.045)	(0.064)	(0.349)	(0.034)	(0.053)	(0.069)	(0.341)
Non-SIB, size quintile 5	0.170***	0.172***	0.281***	2.701***	0.253***	0.261***	0.498***	3.640***
	(0.031)	(0.047)	(0.066)	(0.377)	(0.038)	(0.048)	(0.082)	(0.483)
D-SIB	0.129**	$-$0.044	0.345***	3.816***	0.105	$-$0.060	0.382**	2.838**
	(0.058)	(0.054)	(0.108)	(0.713)	(0.076)	(0.090)	(0.157)	(1.115)
G-SIB	0.072	$-$0.085	0.708***	4.821***	0.007	$-$0.087	0.586*	2.649**
	(0.091)	(0.114)	(0.220)	(1.294)	(0.064)	(0.057)	(0.314)	(1.227)
Controls?	No	No	No	No	No	No	No	No
Year FE?	Yes	Yes	Yes	Yes	Yes	Yes	Yes	Yes
Years	All	All	All	All	2010–2019	2010–2019	2010–2019	2010–2019
Banks	All	All	All	All	All	All	All	All
Nr. banks	155	155	145	155	101	101	101	101
N	2497	2497	2345	2497	927	927	927	927
Mean(dep. var.)	0.37	0.58	0.69	4.81	0.34	0.50	0.69	4.21
SD(dep. var.)	0.19	0.31	0.38	2.61	0.18	0.23	0.37	2.28
Adj. R2	0.15	0.13	0.18	0.25	0.31	0.21	0.30	0.34

Robust standard errors (clustered by bank) in parentheses

Significance: *$$<0.1$$, *$$<0.05$$, ***$$<0.01$$

In panel A, we first decompose the CIR into two subindices: a CIR based on personnel expenses and a CIR based on material expenses. Comparing columns (2) and (3) (or (5) and (7) for the post-crisis period) in Table 10 indicates that for non-SIBs the negative effect of bank size on the CIR is mainly driven by material expenses. This is the case both over the whole sample and over the post-crisis period. In our previous analysis, we found that D-SIBs and G-SIBs tend to have a higher CIR. The results in column (3) indicate that this is mainly due to higher personnel expenses.

When using the EAR as a measure of efficiency, none of the size group coefficients are significant, except for the positive G-SIB indicator. Thus, based on the EAR we do not find evidence for scale efficiencies. However, this may be primarily due to the fact that the EAR, which is based on a bank’s cost relative to its asset base, is highly dependent on the bank’s business model (e.g., Huljak et al. 2019, p. 15). For instance, banks focusing on corporate clients will tend to invest fewer resources in a branch network than banks focusing on retail clients. Thus, banks with higher corporate lending will have lower average costs compared to banks with higher retail lending. Of course, this would also affect the CIR, our primary efficiency measure, although there, income is likely affected by the same factors. Indeed, we find that, unlike for the CIR, the qualitative conclusions when using the EAR as dependent variable are highly sensitive to the addition of other bank characteristics as controls: with controls, we do find significant and large-scale effects for non-SIBs (see Additional file 1: Appendix Table A.3).

Turning to profitability (panel B), the results using NOI/TA are very similar to those using ROA. This is not surprising, since the two measures only differ marginally.^34^ The positive relationship between bank size and profitability for non-SIBs is confirmed if we use RORWA and ROE as alternative profitability measures. The effect is more pronounced over the post-crisis period. The RORWA pattern implies that higher returns for larger banks are not driven by higher risk-taking.

Notably, for both RORWA and ROE the D-SIB and G-SIB dummies are positive and significant in the post-GFC period, which is not the case for ROA. While the point estimates for D-SIBs are still lower than for the largest non-SIBs, at least for RORWA the G-SIB estimate is larger than for all non-SIB groups, although it is not very precisely estimated. Mechanically, these results are driven by lower risk density (RWA over total assets) and lower leverage ratio (capital over assets) for SIBs than for non-SIBs. Indeed, the mean of RWA over total assets is substantially higher for non-SIBs (55%) than for SIBs (39%) (see Additional file 1: Appendix Table A.1). The mean of capital over assets is also substantially higher for non-SIBs (8%) than for SIBs (5%).

The RORWA result for SIBs would imply that (G-)SIBs are actually more profitable than the largest non-SIBs if we use a profitability measure that considers the banks’ risk structure, suggesting that scale economies extend even to the largest banks. However, we should interpret the RORWA results for G-SIBs with caution. While it is certainly plausible that G-SIBs have lower risk density than the non-SIBs in our sample, the measured differences likely also reflect that the G-SIBs use internal models to calculate RWA, which may result in lower RWA than the standardized approach of the Basel Committee on Banking Supervision used by the non-SIBs.^35^

## Discussion of results

For a large sample of non-systemically important Swiss banks, we find strong evidence for scale economies. Non-SIBs can substantially increase their efficiency and profitability by increasing in size: a one-standard-deviation increase in log(Total Assets) is associated with a reduction in the CIR of about 0.2 standard deviations and an increase in ROA of about 0.4 standard deviations. The magnitude of these scale effects is comparable to Kovner et al. (2014)’s findings for US banks over 2001–2012.^36^ Alternative outcome measures produce similar implications. Moreover, using an instrumental variables approach for a subset of geographically restrained banks, we find that the effect of size on efficiency and profitability is likely causal.

Our results indicate much stronger scale efficiencies since 2010 than in the years prior to the GFC. This implies that bank size has become more important for bank efficiency and profitability over recent years, at least for non-SIBs. This finding is in line with the growing importance of digital technologies in banking. The adoption of these technologies is associated with potentially large economies of scale: larger banks can spread their IT expenses over a larger asset base. The stronger size effects since 2010 could also be related to the low interest rate environment, which has coincided with a compression of bank interest margins. These developments may have forced banks to take measures to preserve income margins, e.g., by raising more fee income. Basten and Mariathasan (2020) show that banks with more market power are more able to do so. Thus, to the extent that size coincides with more market power (perhaps beyond our control variable based on local HHI), the stronger scale effects in recent years might partly reflect this channel as well.

The evidence for scale economies becomes stronger by adding different controls for bank characteristics to the CIR regression, such as the bank’s business model and revenue structure, the bank’s risk profile and market concentration. This implies that larger banks tend to have characteristics that are associated with lower efficiency. Not controlling for these characteristics understates the estimated relationship between bank size and efficiency. Our results indicate that banks with more capital are relatively more efficient and more profitable. We do not attach a causal interpretation to this relationship. It may be the case that efficient and profitable banks build up capital by not paying out all of their profits to their shareholders. At least, the results show that good capitalization and high efficiency and profitability are compatible.

The question of whether the largest (systemically important) banks also exhibit scale efficiencies is more challenging. First, the largest Swiss banks, and in particular the two G-SIBs UBS and Credit Suisse, have rather different characteristics than the non-SIBs. Thus, it is difficult to know to what extent observed differences in efficiency or profitability can be explained by size itself or by differences in other bank characteristics. Second, the sample of SIBs is limited to five banks in Switzerland. Nevertheless, we also use different approaches to analyze scale efficiencies for SIBs to the extent possible. D-SIBs and especially G-SIBs display higher CIRs and lower ROAs than the largest non-SIBs, especially in the post-crisis period. This raises the question whether the post-crisis results might be driven by increased costs due to regulation introduced after the GFC, in particular higher capital requirements for SIBs. Our results do not appear supportive of this hypothesis. First, the results suggest that at least to some extent, the differences in efficiency and profitability between SIBs and non-SIBs can be “explained away” by adding the bank characteristics as controls. Rather than regulation, certain aspects of G-SIBs’ business models, in particular their international diversification or the relatively high trading asset share, may make it difficult for them to realize scale efficiencies. Second, the within-bank results show that when looking after the GFC, there is no evidence for scale economies within D-SIB and G-SIB groups, although G-SIBs have substantially higher CIRs and lower ROAs. Third, we find that higher capitalization is not detrimental for efficiency and profitability.

We interpret the documented empirical patterns—efficiency and profitability measures increasing in bank size—as evidence for economies of scale. There may be alternative explanations in addition to economies of scale. First, large banks may operate closer to their efficient frontier on average, i.e., have greater X-efficiency. Scale efficiency and X-efficiency are closely related: total cost efficiency is the product of scale and X-efficiencies (see Berger and Mester , 1997, p. 926). Since we do not estimate bank cost functions, we do not differentiate between the two efficiency channels. Second, higher bank profitability may be the result of market power.^37^ In our analysis, we include an HHI capturing the mortgage market concentration as a proxy for a bank’s market power. Our results indicate that banks operating in more concentrated markets tend to have a higher level of profitability. Third, large banks may have greater bargaining power vis-á-vis their suppliers (i.e., buyer power) or employees (i.e., monopsony power). If cost savings are due to bargaining power effects, this implies differences in the allocation of rents between the banks and their suppliers and employees, rather than higher bank productivity. Overall, regardless of the exact source of the effects, our results indicate that within non-SIBs, larger banks are more efficient and more profitable than smaller banks.

## Conclusion

The banking sector currently has to deal with important challenges in the form or reduced profitability driven by low interest rates and increased competition from FinTech and BigTech firms. The COVID-19 crisis adds to those pre-existing challenges and will put further restructuring pressure on the banking sector. In such an environment, we expect that efficiency and profitability will take center stage. Our evidence for scale economies suggests that there is a substantial potential for increasing efficiency and profitability by increasing in size for most Swiss banks.

Our evidence on scale economies is more conclusive for non-SIBs than for the largest banks. The Swiss G-SIBs have substantially higher CIRs and lower ROAs than non-SIBs. However, G-SIBs have rather different characteristics than the non-SIBs. Thus, it is difficult to know to what extent observed differences in efficiency or profitability can be explained by size itself or by differences in other bank characteristics. Our results suggest that certain aspects of G-SIBs’ business models, in particular their international diversification or the relatively high trading asset share, may make it difficult for them to realize scale efficiencies. To gain more insights about scale efficiencies for G-SIBs, it would be necessary to complement our analysis with an international sample covering G-SIBs with a comparable business model. Such an analysis would also make it possible to shed light on potential efficiency effects of consolidation among these banks, or conversely, of reducing their scale and scope.

Finally, in our analysis we focus on scale effects on cost efficiency and profitability without trying to disentangle the contributions of the two main functions of banks, which are brokerage and qualitative asset transformation. In particular, it would be interesting to study in future work whether there are economies of scale specifically in banks’ financial risk management.

## Supplementary Information

**Additional file 1**. Online Appendix.

## Data Availability

The datasets used in this study are not publicly available due to their confidential nature. The authors will facilitate access to the data upon reasonable request and only with permission of the Swiss National Bank. The Stata codes used in the analysis are available from the authors.

## Abbreviations

2SLS: Two-stage least squares
CHF: Swiss franc
CIR: Cost-to-income ratio
D-SIB: Domestically focused systemically important bank
EAR: Expense assets ratio
ECB: European Central Bank
GDP: Gross domestic product
GFC: Global financial crisis
G-SIB: Global systemically important bank
HHI: Herfindahl–Hirschman index
IV: Instrumental variable
NOI: Net operating income
OLS: Ordinary least squares
Q: Quintile
ROA: Return on assets
ROE: Return on equity
RORWA: Return on risk-weighted assets
RWA: Risk-weighted assets
SIB: Systemically important bank
SNB: Swiss National Bank
TA: Total assets
TBTF: Too big to fail
US: United States
WM: Wealth management
ZKB: Zürcher Kantonalbank

## Footnotes

1 For example, Fu and Mishra (2020) estimate an increase in finance app downloads of around 30 percent during the peak of the pandemic. In Switzerland, survey evidence indicates rapid adoption of mobile payment solutions and neobank apps, especially among younger consumers (https://www.moneyland.ch/en/swiss-payments-survey-2021).

2 The 26 cantons are the administrative subdivisions of Switzerland. Twenty-four of them have cantonal banks; two cantons (Appenzell Ausserrhoden and Solothurn) sold or privatized their cantonal banks in the mid-1990s, before the start of our sample period. Out of the 24 banks, 21 feature a full guarantee of bank liabilities by their respective canton. We also separately study whether these guarantees may affect our estimated scale effects in the non-SIB sample, finding that cantonal banks have relatively better CIRs but not ROAs than would be expected based on their size.

3 See https://www.bankingsupervision.europa.eu/press/pr/date/2021/html/ssm.pr210112 920b511a1c.en.html.

4 Berger and Humphrey (1997) provide a detailed review of the early literature, documenting studies with data from various countries.

5 Dietrich and Wanzenried (2014) confirm the negative relationship between the CIR and ROA in a further study of the determinants of bank profitability with a sample of banks from 118 countries over the period from 1998 to 2012.

6 See Financial Stability Board (2021) for a recent overview of TBTF issues and the effects of post-crisis reforms.

7 For a description of the structure of the Swiss banking sector, see Swiss National Bank (2021), pp. 17–19. For detailed statistics concerning the Swiss banking sector, see Swiss National Bank (2020).

8 See Annex 2 in Financial Stability Board (2014) for a detailed discussion.

9 The Raiffeisen group consists of over 200 independent bank cooperatives; however, we observe it in our data at the group level only.

10 We use nonconsolidated data for Bank Coop/Cler and Sparkasse Engelberg. These banks are part of their parent banks Basler Kantonalbank and Sparkasse Schwyz. To avoid double counting, we use nonconsolidated data for the latter banks as well.

11 The aggregate ROA for US banks has been between 0.5 and 1.3% in most years over the last decade; see https://www.federalreserve.gov/publications/2020-may-supervision-and-regulation-report-banking-system-conditions.htm.

12 See https://www.economist.com/leaders/2019/04/06/fixing-europes-zombie-banks.

13 See Swiss National Bank (2020), pp. 22–25. In aggregate, the securities in custody accounts at Swiss banks are about twice as large as the combined total assets of the banks (Swiss National Bank 2020, p. 5). Moreover, the globally active Swiss banks increasingly offer fiduciary investments to their customers as an alternative to on-balance-sheet deposits. However, the aggregate value of fiduciary transactions in the Swiss banking sector relative to banks’ total assets is only about 6%.

14 The Fed uses return on equity and return on average assets as measures of profitability in its regular analysis of financial performance of the banking industry. See https://www.federalreserve.gov/publications/2020-november-supervision-and-regulation-report-banking-system-conditions.htm.

15 We classify these banks as SIBs for the entire period during which they are in the sample; that is, not just once they were officially designated as SIBs.

16 SIBs are subject to higher capital and liquidity requirements as well as specific requirements for resolvability in a crisis.

17 Corresponding time series for the alternative metrics discussed above are shown in Additional file 1: Appendix Figure A.3. They paint a largely similar picture, except that the EAR has decreased steadily since the GFC, and RORWA has stayed flat (unlike ROA or ROE). The difference between the EAR and the CIR is caused by the denominator, not the numerator: the EAR decreases over time because assets have increased relative to income.

18 We assume a linear relationship between log(Total Assets) and CIR (or ROA) for simplicity. Additional file 1: Appendix Figure A.4 shows that this appears to be a good approximation for the CIR, while for ROA, the relationship with size appears to be flat in the bottom half of the size distribution. Below, we report results from a more flexible approach where banks are divided into size groups.

19 However, there is now much less variation in the right-hand-side variable: within bank, one standard deviation in residual log(Total Assets) corresponds to about 0.13, and after adding year fixed effects, the residual variation further decreases by about half, to 0.06.

20 To be clear, it is more likely that return on equity (ROE) decreases in capital ratios, given the “mechanical” link between the two measures (ROE has capital in the denominator, while capital/assets have it in the numerator). We analyze ROE as an alternative outcome variable in Sect. 4.4.

21 The networks identified in the bank statistics we are using are Entris, Clientis and Esprit; a bank can be a member of more than one (and in fact, Clientis banks are a subset of Entris banks). Of the 89 banks in the sample in 2019, 47 are members of at least one of these networks, 24 are cantonal banks, and 18 are neither member of a network nor cantonal banks.

22 Several banks which are network members are cooperatives/mutual banks, which by itself might affect profitability or efficiency. Since we do not have systematic data on ownership structure of our sample banks, we cannot disentangle governance from network effects.

23 A number of papers have analyzed the effects of changes in size from mergers or acquisitions (e.g., Cornett et al. 2006); however, these events are themselves usually not exogenous and also have direct costs.

24 There is no general legal restriction to prevent cantonal banks from operating outside their own canton, but cantonal laws typically stipulate that cantonal bank operations are focused on the home canton. Traditionally, cantonal banks did not seem willing to compete outside their respective territories (e.g., Swiss Competition Commission 1998, p. 302). In recent years, however, cantonal banks have increased their operations outside their canton (Krähenbül 2019).

25 Additional file 1: Appendix Figure A.5 shows the strong correlation between cantonal bank size as of 2019 (the last year in our sample) and the canton’s population in 1995. Also, we note that using contemporaneous population levels rather than those from 1995 leaves the instrumented coefficient on log(Total Assets) essentially unchanged.

26 Since the regressions in this table only feature 24 clusters, the cluster-robust standard errors we report are potentially unreliable. “Wild bootstrap” standard errors (based on the Stata package of Roodman et al. , 2019) yield p values for Log(Total Assets) of 0.07 in column (4) and 0.01 in column (8) of panel B.

27 To provide a sense of the magnitudes, the population share with tertiary education, which we measure as of 2019 (but is likely slow moving), ranges from 0.22 to 0.46. Not including this variable in the regression leaves the coefficients on log(Total Assets) essentially unchanged. We have also experimented with adding additional canton-level controls, such as median income, female labor force participation, or the population share of foreigners. These variables were not statistically significant and adding them left the instrumented coefficient on log(Total Assets) qualitatively unchanged.

28 For the full sample period, we do not include the last three control variables, as in column (5) of these tables; for the post-crisis period, we do include them, as in column (7).

29 The finding of higher CIRs for the D-SIBs than the largest non-SIBs is not driven by any particular D-SIB. For ROA, on the other hand, one D-SIB is known to have had particularly low values in recent years (PostFinance, see Swiss National Bank , 2021, p. 29). The other two D-SIBs on average exhibit only slightly lower ROAs than the largest non-SIBs without controls, and slightly higher ROAs with control variables added to the regression.

30 Since this is admittedly a bit complicated: $[-15.479 -(-17.203)]-[14.368-(-10.676)] = -23.3$.

31 Given the small number of banks in these regressions, we do not cluster standard errors, but simply report robust standard errors, which should be interpreted with caution.

32 Corresponding regressions with controls are reported in Additional file 1: Appendix Table A.3.

33 The time-series evolution of these metrics is shown in Additional file 1: Appendix Figure A.3.

34 We have also considered a version of ROA where liquid assets are subtracted from the denominator, given that SNB sight deposits in the banking system overall have increased substantially in recent years along with the Swiss National Bank’s balance sheet expansion. Using this alternative metric produces very similar results as when we use the “usual” ROA (not reported); directionally, the evidence for scale economies strengthens.

35 The differences in RWA density between G-SIBs and non-SIBs in our data are very large. In fact, over 2010–2019, the *highest* RWA density value among G-SIBs is about equal to the *lowest* RWA density reported by any non-SIB. To restore credibility in the calculation of RWA, the Basel Committee agreed on a 72.5% output floor based on the Committee’s revised standardized approaches. A monitoring report shows that this floor has an impact on the minimum required capital for a group of internationally active banks, which indicates that at least for some banks the risk weights used in the internal models are indeed substantially lower than those used in the standardized approach (see Bank for International Settlements 2017).

36 The coefficients of bank size in our regressions of the CIR on log(Total Assets) and controls in Table 3 are similar to the coefficients in Kovner et al. (2014), Table 4, p. 13. They use the “efficiency ratio” as dependent variable, but that measure is almost identical to the CIR in our data.

37 Drechsler et al. (2017) show that market power in deposit markets allows banks to increase deposit spreads after the central bank increases the funds rate.
