# Supplementary material for: Scale effects on efficiency and profitability in the Swiss banking sector
Source: Swiss J Econ Stat. 2022 May 9;158(1):12. doi: 10.1186/s41937-022-00091-7 (PMC9080912; doi:10.1186/s41937-022-00091-7)
Supplement: Supplementary file 1 — Additional file 1. Online Appendix. [file 41937_2022_91_MOESM1_ESM.pdf]

Online Appendix for
"Scale Effects on Efficiency and Profitability in the Swiss Banking Sector"
by Marc Blatter and Andreas Fuster

A Additional results

Table A.1: Descriptive statistics, by SIB status

	Non-SIBs				SIBs			
	Mean	St. Dev.	Min	Max	Mean	St. Dev.	Min	Max
Cost/Income Ratio (%)	53.71	9.95	32.37	87.25	66.53	9.99	50.72	87.25
Return on Assets (%)	0.37	0.19	-0.06	1.33	0.41	0.30	-0.06	1.33
Personnel Cost/Income (%)	30.07	6.36	17.18	56.92	44.49	8.70	26.34	56.92
Material Cost/Income (%)	23.64	6.57	11.51	44.24	21.81	6.19	13.13	44.24
Expense/Asset Ratio (%)	1.08	0.33	0.59	2.94	1.68	0.62	0.83	2.94
Net Operating Income/Assets (%)	0.58	0.30	-0.62	1.75	0.45	0.40	-0.62	1.75
Return on Risk-Wtd. Assets (%)	0.67	0.34	-0.09	2.63	1.13	0.76	-0.09	2.63
Return on Equity (%)	4.67	2.25	-1.73	18.07	7.98	5.97	-1.73	18.07
Log(Total Assets)	20.64	1.74	15.40	24.60	26.42	1.11	24.74	28.48
Deposits/Assets (%)	64.30	8.77	40.23	90.22	55.00	15.36	39.34	90.22
Mortgages/Assets (%)	75.25	9.72	10.08	88.03	36.75	26.94	10.08	82.45
Trading/Assets (%)	0.33	1.24	0.00	15.72	9.76	8.14	0.00	20.85
Net Int. Inc./Op. Inc. (%)	79.38	13.43	21.86	101.53	48.61	22.89	21.86	101.53
Commission Inc./Op. Inc. (%)	14.78	10.57	0.00	63.21	41.09	18.44	6.47	63.21
Trading Inc./Op. Inc. (%)	3.80	3.67	-5.62	22.52	11.26	7.35	-5.62	22.52
Capital/Assets (%)	8.08	1.99	3.59	14.84	5.10	1.20	3.59	7.39
RWA/Assets (%)	55.17	7.57	37.01	81.99	39.44	12.16	25.31	63.98
Domestic/Total Assets (%)	98.12	3.76	47.46	100.00	56.61	32.23	19.31	99.96
HHI of Mtg Holdings (/1000)	7.71	2.29	0.93	10.00	2.89	3.01	0.82	10.00
Avg. Local HHI (/1000)	1.96	0.55	1.12	3.64	1.99	0.20	1.48	2.53

Table A.2: Non-SIB regressions, controlling for cantonal bank and network membership status

	(1) CIR	(2) CIR	(3) CIR	(4) CIR	(5) ROA	(6) ROA	(7) ROA	(8) ROA
Log(Total Assets)	-0.796	-2.901***	-1.162*	-3.762***	0.021**	0.019**	0.027***	0.037***
	(0.541)	(0.514)	(0.694)	(0.897)	(0.009)	(0.009)	(0.010)	(0.011)
Cantonal Bank	-5.088**	-3.265*	-8.529***	-5.605*	0.018	-0.004	0.090**	-0.021
	(2.322)	(1.831)	(3.004)	(3.251)	(0.047)	(0.038)	(0.040)	(0.044)
Banking Network Member	-3.264*	-3.628**	-2.949	-4.484***	-0.091***	-0.054**	-0.065**	-0.060**
	(1.838)	(1.463)	(2.389)	(1.686)	(0.031)	(0.021)	(0.030)	(0.026)
Year FE?	Yes	Yes	Yes	Yes	Yes	Yes	Yes	Yes
Controls?	No	Yes	No	Yes	No	Yes	No	Yes
Years	All years	All years	2010-2019	2010-2019	All years	All years	2010-2019	2010-2019
Nr. banks	148	139	96	96	148	139	96	96
N	2390	2242	881	880	2390	2242	881	880
Mean(dep. var.)	53.71	54.14	57.86	57.89	0.37	0.37	0.34	0.34
SD(dep. var.)	9.95	9.88	9.47	9.44	0.19	0.19	0.17	0.17
Adj. R2	0.22	0.35	0.22	0.40	0.19	0.45	0.35	0.62
Adj. R2 (within)	0.08	0.25	0.23	0.40	0.16	0.43	0.35	0.62

Robust standard errors (clustered by bank) in parentheses.

Significance: $^{*} < 0.1$, $^{*} < 0.05$, $^{***} < 0.01$.

Note: Regressions correspond to a subset of those reported in Tables 2 to 4, but controlling for dummies = 1 if a bank is a cantonal bank or is member of a network (Entris, Clientis, Esprit). In regressions with other controls, the full set of controls as in columns (5) and (7) of Tables 3 and 4 are used.

Table A.3: Alternative measures for efficiency and profitability: regressions with bank characteristic controls

	(1)<br>CIR	(2)<br>CIR_{mat}	(3)<br>CIR_{pers}	(4)<br>EAR	(5)<br>CIR	(6)<br>CIR_{mat}	(7)<br>CIR_{pers}	(8)<br>EAR
Non-SIB, size quintile 2	-5.314***	-3.874***	-1.436	-0.132***	-4.742**	-3.257**	-1.594	-0.062*
	(1.346)	(0.889)	(1.077)	(0.034)	(1.835)	(1.356)	(1.615)	(0.037)
Non-SIB, size quintile 3	-5.610***	-5.550***	-0.099	-0.162***	-4.899**	-5.151***	0.144	-0.189***
	(1.778)	(1.032)	(1.329)	(0.043)	(2.371)	(1.649)	(1.992)	(0.050)
Non-SIB, size quintile 4	-9.402***	-8.803***	-0.635	-0.322***	-11.484***	-12.218***	0.630	-0.342***
	(1.812)	(1.081)	(1.392)	(0.054)	(2.563)	(1.652)	(2.042)	(0.057)
Non-SIB, size quintile 5	-13.489***	-11.391***	-2.124	-0.408***	-17.375***	-14.026***	-3.498	-0.403***
	(1.976)	(1.126)	(1.536)	(0.055)	(2.813)	(1.801)	(2.340)	(0.058)
D-SIB	-6.949**	-10.295***	3.357	-0.289**	-8.985**	-13.446***	4.387	-0.201**
	(2.750)	(1.823)	(2.633)	(0.127)	(3.836)	(3.713)	(3.979)	(0.096)
G-SIB	-5.457	-14.605***	7.878	0.292*	-12.049	-23.190*	10.111	1.234***
	(5.706)	(2.501)	(4.911)	(0.165)	(14.525)	(13.137)	(10.963)	(0.372)
Controls?	Yes	Yes	Yes	Yes	Yes	Yes	Yes	Yes
Year FE?	Yes	Yes	Yes	Yes	Yes	Yes	Yes	Yes
Years	All	All	All	All	2010-2019	2010-2019	2010-2019	2010-2019
Banks	All	All	All	All	All	All	All	All
Nr. banks	145	145	145	145	101	101	101	101
N	2343	2343	2343	2343	926	926	926	926
Mean(dep. var.)	54.68	23.75	30.92	1.10	58.43	24.96	33.47	1.00
SD(dep. var.)	10.20	6.57	7.10	0.36	9.66	7.13	6.79	0.30
Adj. R2	0.37	0.31	0.43	0.70	0.36	0.40	0.39	0.75

	(1)<br>ROA	(2)<br>NOI/TA	(3)<br>RORWA	(4)<br>ROE	(5)<br>ROA	(6)<br>NOI/TA	(7)<br>RORWA	(8)<br>ROE
Non-SIB, size quintile 2	-0.015	0.005	-0.026	-0.186	-0.006	0.008	-0.021	-0.154
	(0.020)	(0.033)	(0.037)	(0.259)	(0.019)	(0.036)	(0.039)	(0.264)
Non-SIB, size quintile 3	-0.000	0.008	-0.006	-0.133	-0.011	-0.003	-0.027	-0.297
	(0.023)	(0.037)	(0.041)	(0.316)	(0.022)	(0.038)	(0.046)	(0.321)
Non-SIB, size quintile 4	0.038	0.084**	0.085*	0.596*	0.055*	0.098*	0.112*	0.617
	(0.026)	(0.041)	(0.047)	(0.356)	(0.031)	(0.051)	(0.061)	(0.425)
Non-SIB, size quintile 5	0.126***	0.168***	0.246***	1.770***	0.202***	0.265***	0.408***	2.470***
	(0.031)	(0.048)	(0.057)	(0.455)	(0.036)	(0.053)	(0.071)	(0.565)
D-SIB	0.194***	0.083	0.345**	3.087***	0.145*	0.049	0.298*	1.460
	(0.071)	(0.088)	(0.141)	(0.997)	(0.076)	(0.116)	(0.160)	(1.191)
G-SIB	0.232	0.257	0.577*	4.710	0.438	0.674**	1.155*	4.076
	(0.176)	(0.218)	(0.315)	(2.891)	(0.282)	(0.338)	(0.595)	(3.852)
Controls?	Yes	Yes	Yes	Yes	Yes	Yes	Yes	Yes
Year FE?	Yes	Yes	Yes	Yes	Yes	Yes	Yes	Yes
Years	All	All	All	All	2010-2019	2010-2019	2010-2019	2010-2019
Banks	All	All	All	All	All	All	All	All
Nr. banks	145	145	145	145	101	101	101	101
N	2343	2343	2343	2343	926	926	926	926
Mean(dep. var.)	0.37	0.58	0.69	4.78	0.34	0.50	0.69	4.19
SD(dep. var.)	0.19	0.31	0.37	2.62	0.17	0.23	0.37	2.24
Adj. R2	0.43	0.36	0.40	0.34	0.58	0.41	0.53	0.46

Robust standard errors (clustered by bank) in parentheses. Significance: $^{*} < 0.1$, $^{*} < 0.05$, $^{***} < 0.01$.

Figure A.1: Distribution of managed securities (relative to total assets) across bank size groups

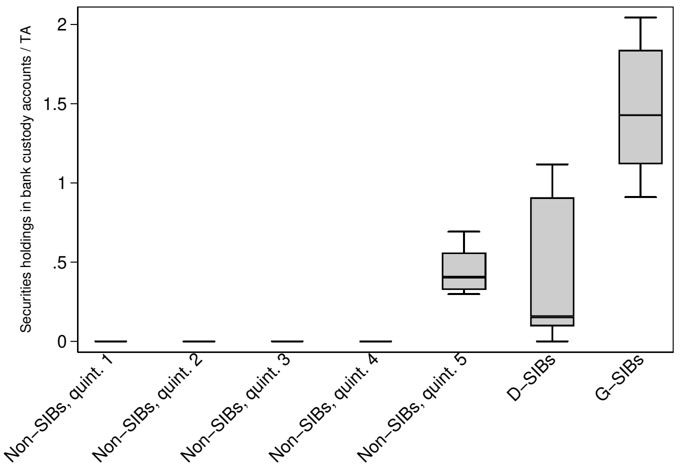

Note: Box plots for pooled sample period 2010-2019. Boxes show 25th, 50th, and 75th percentile; whiskers show 10th and 90th percentile. Size quintiles for non-SIBs are defined by year.

Figure A.2: Evolution of bank characteristics in sample over time

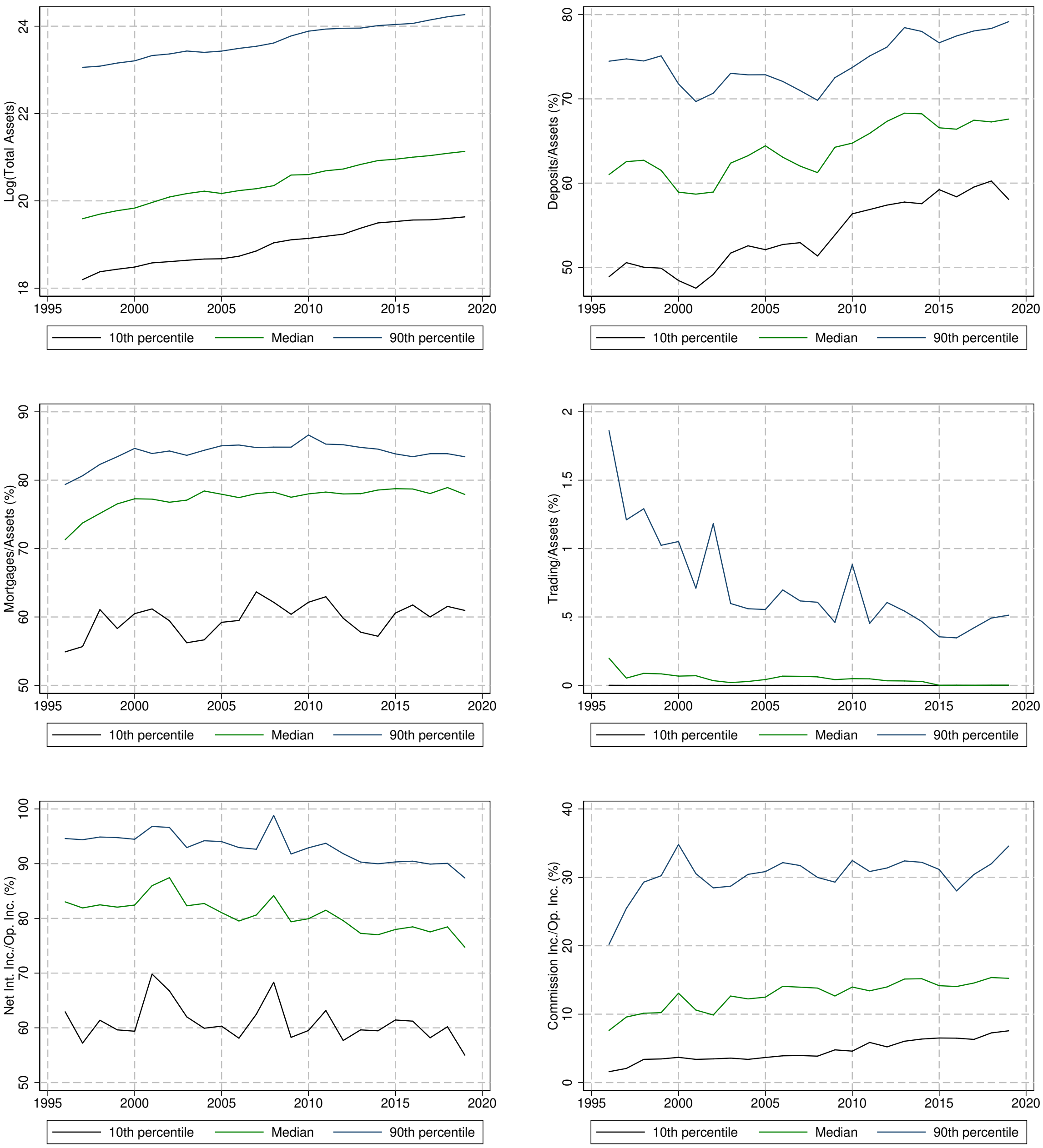

Notes: Figure shows the evolution of median, 10th percentile, and 90th percentile across banks in our sample of various bank characteristics used as control variables in some of the regressions in the paper. Data source: SNB.

Figure A.2: Evolution of bank characteristics in sample over time (continued)

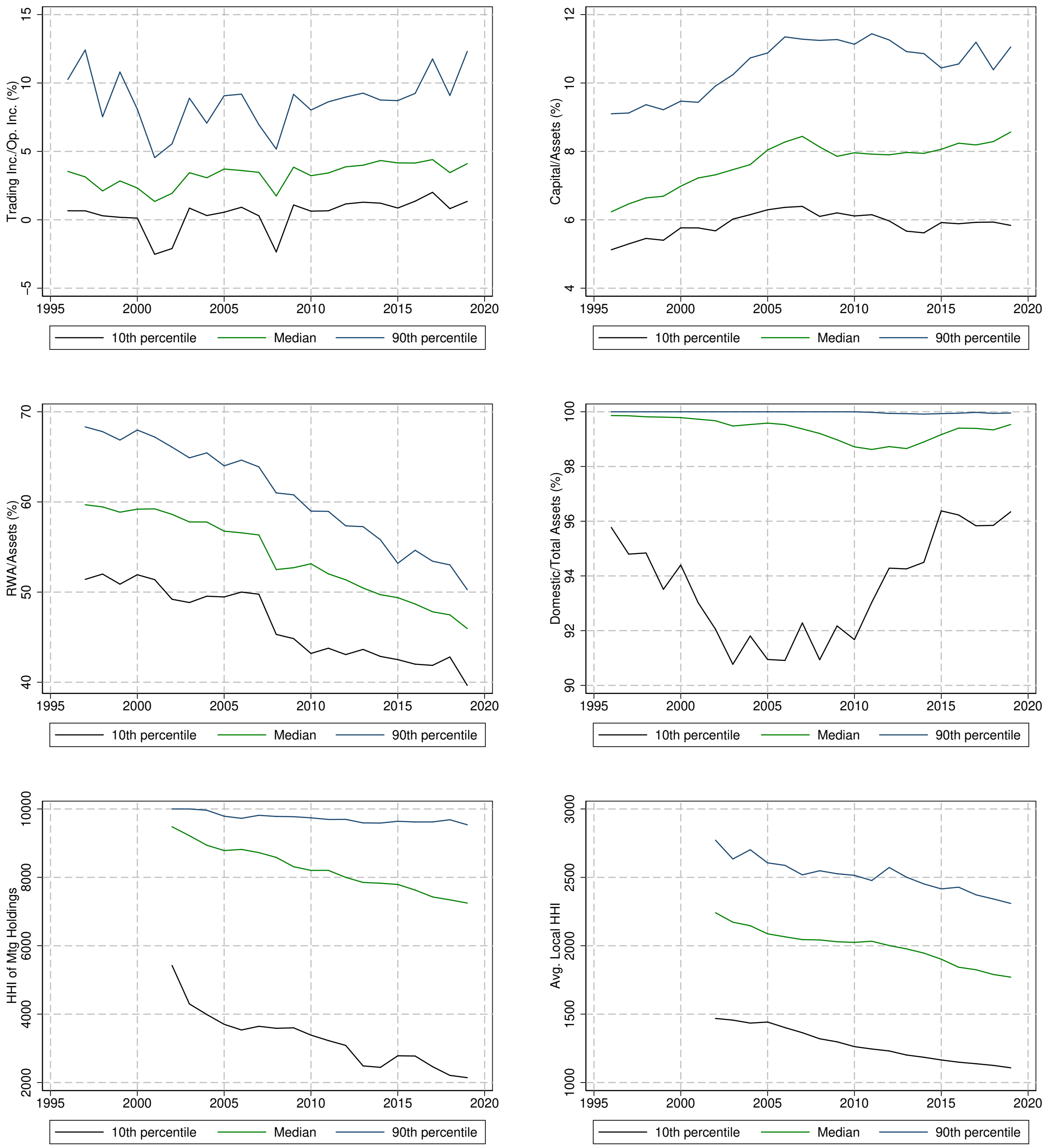

Notes: Figure shows the evolution of median, 10th percentile, and 90th percentile across banks in our sample of various bank characteristics used as control variables in some of the regressions in the paper. Data source: SNB.

Figure A.3: Evolution of alternative efficiency and profitability metrics in sample over time

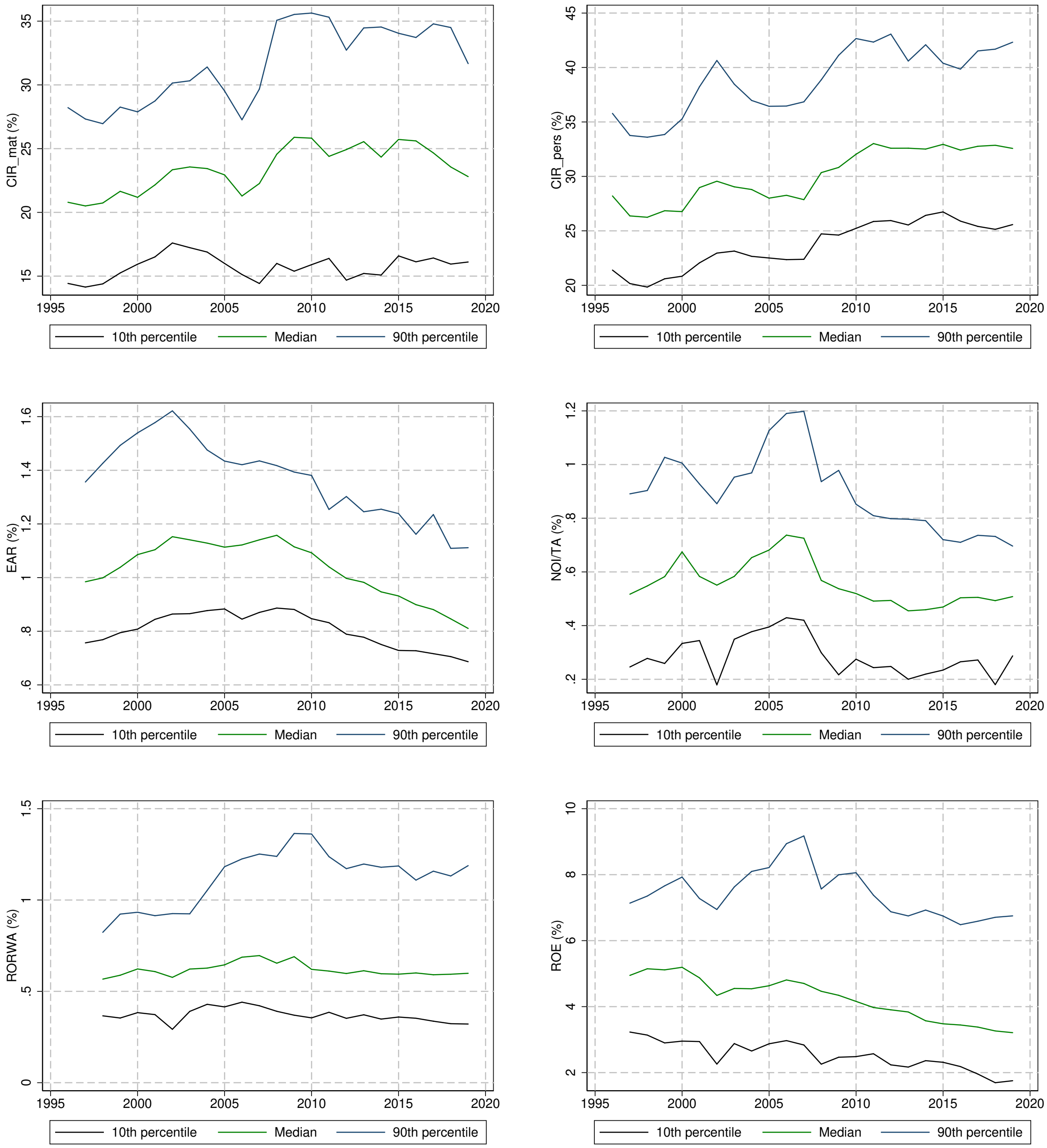

Notes: Figure shows the evolution of median, 10th percentile, and 90th percentile across banks in our sample of various alternative outcome measures discussed in Section 4.4. Data source: SNB.

Figure A.4: Evidence on (non)linearity of relationship between log(Total Assets) and efficiency/profitability

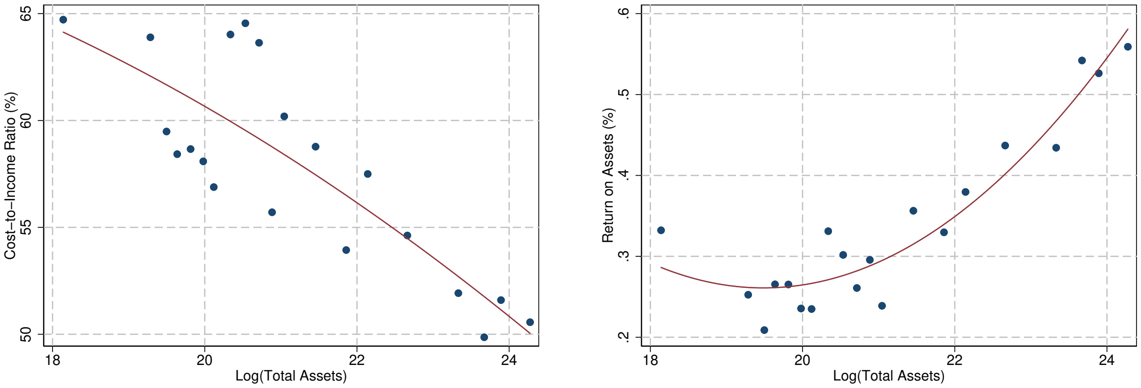

Note: Binned scatter plots with quadratic fit line. In both panels, only non-SIBs are included, the sample period is 2010-2019, and year fixed effects are absorbed.

Figure A.5: Total assets of cantonal banks in 2019 vs. population of their home canton in 1995

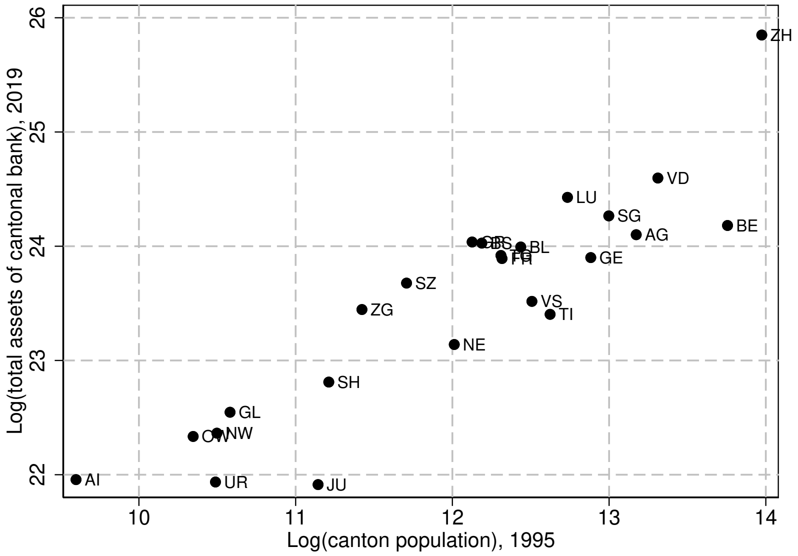

Note: See https://en.wikipedia.org/wiki/Data_codes_for_Switzerland#Cantons for the list of canton codes used in the chart.

B Efficiency, size and exit

Over the course of our sample period, 60 banks exit the sample.[2] Of those exits, 22 occurred through mergers, while 38 banks were acquired. This section features a brief analysis linking exits to bank size and efficiency.

Specifically, we estimate a linear probability model

$$exit_{i,t} = \alpha_t + \beta_1 log(TotalAssets)_{i,t-2} + \beta_2 CIR_{i,t-2} + \Gamma X_{i,t-2} + \varepsilon_{i,t}. \quad (9)$$

where $exit_{i,t}$ is a binary variable that equals 100 if our last observation for bank i occurs in year t, meaning that in the following year it either merges with or is acquired by another bank. Our two key control variables are Log(Total Assets) and CIR, which we lag by two years in order to account for possible changes over the two years immediately preceding the exit. In some specifications we further add the additional control variables that are also featured in the main text, also lagged by two years. We also feature year fixed effects in order to account for common factors affecting the propensity to exit.

Results are shown in Table A.4. The results indicate that smaller banks and banks with higher CIRs (meaning lower efficiency) are more likely to exit in general or to be acquired. This is in line with Assaf et al. (2019), who find that cost efficiency reduces bank failure probabilities.[3] In terms of economic magnitude, a 10 percentage point higher CIR, corresponding roughly to a move from the median to the 90th percentile within a year (see Figure 3) increases the annual probability of exit by about 1.3 percentage points, or more than half of the unconditional probability in the sample. The effects of size are somewhat more modest: doubling in size reduces the annual exit probability by about 0.5-0.6 percentage points. The probabilities of exiting the sample as part of a merger are not significantly affected by either the CIR or size.

Table A.4: Linear probability model of bank exits

	(1)<br>Exit	(2)<br>Exit	(3)<br>Acquired	(4)<br>Acquired	(5)<br>Merger	(6)<br>Merger
Cost/Income Ratio	0.130***	0.146***	0.113***	0.151***	0.016	-0.005
	(0.040)	(0.053)	(0.036)	(0.046)	(0.017)	(0.026)
Log(Total Assets)	-0.636***	-0.527**	-0.594***	-0.350*	-0.042	-0.177
	(0.136)	(0.233)	(0.118)	(0.190)	(0.078)	(0.139)
Year FE?	Yes	Yes	Yes	Yes	Yes	Yes
Other controls?	No	Yes	No	Yes	No	Yes
Nr. banks	141	141	141	141	141	141
N	2197	2197	2197	2197	2197	2197
Mean(dep. var.)	2.05	2.05	1.41	1.41	0.64	0.64
Adj. R2	0.03	0.03	0.03	0.03	0.01	0.01

Robust standard errors (clustered by bank) in parentheses.

Significance: $^{*} < 0.1$, $^{*} < 0.05$, $^{***} < 0.01$.

[2] In Figure 1, the number of banks in the sample decreases by 63 observations. Some banks leave the sample because they were reclassified or their reporting requirement ends; we do not classify these events as exits for the purpose of the analysis in this section. Also of note, 14 exits occurred by 1998, i.e., very early in our sample period.

[3] Wheelock and Wilson (2000) also find that banks with low earnings and high managerial inefficiency are more likely to fail than other banks. However, they find that inefficient banks are less attractive take-over candidates.

References

Assaf, A. G., A. N. Berger, R. A. Roman, and M. G. Tsionas (2019): “Does efficiency help banks survive and thrive during financial crises?” *Journal of Banking & Finance*, 106, 445–470.

Wheelock, D. C. and P. W. Wilson (2000): “Why do banks disappear? The determinants of US bank failures and acquisitions,” *Review of Economics and Statistics*, 82, 127–138.
